# Anatomy, taphonomy, and phylogenetic implications of a new specimen of *Eolambia caroljonesa* (Dinosauria: Ornithopoda) from the Cedar Mountain Formation, Utah, USA

**DOI:** 10.1371/journal.pone.0176896

**Published:** 2017-05-10

**Authors:** Andrew T. McDonald, Terry A. Gates, Lindsay E. Zanno, Peter J. Makovicky

**Affiliations:** 1 Saint Louis Science Center, Saint Louis, Missouri, United States of America; 2 Department of Biological Sciences, North Carolina State University, Raleigh, North Carolina, United States of America; 3 North Carolina Museum of Natural Sciences, Raleigh, North Carolina, United States of America; 4 Section of Earth Sciences, Field Museum of Natural History, Chicago, Illinois, United States of America; Hebrew University, ISRAEL

## Abstract

**Background:**

*Eolambia caroljonesa* is the most abundant dinosaur in the lower Cenomanian Mussentuchit Member of the Cedar Mountain Formation of Utah, and one of the most completely known non-hadrosaurid iguanodontians from North America. In addition to the large holotype and paratype partial skulls, copious remains of skeletally immature individuals, including three bonebeds, have been referred to *E*. *caroljonesa*. Nevertheless, aspects of the postcranial anatomy of this taxon, particularly the pelvic girdle, have remained ambiguous due to the lack of associated postcranial material of larger, more mature individuals.

**Methodology/Principal findings:**

Here we describe a recently discovered associated partial postcranial skeleton of a large *Eolambia caroljonesa*. This specimen, FMNH PR 3847, provides new anatomical data regarding the vertebral column and pelvic girdle, supplementing previous diagnoses and descriptions of *E*. *caroljonesa*. A new phylogenetic analysis incorporating information from FMNH PR 3847 places *E*. *caroljonesa* as a basal hadrosauromorph closely related to *Protohadros byrdi* from the Cenomanian Woodbine Formation of Texas. Histological analysis of FMNH PR 3847 reveals that it represents a subadult individual eight to nine years of age. Taphonomic analysis indicates that FMNH PR 3847 was preserved in a crevasse splay deposit, along with an unusual abundance of small crocodylomorph material.

**Conclusions/Significance:**

FMNH PR 3847 provides a wealth of new morphological data, adding to the anatomical and systematic characterization of *Eolambia caroljonesa*, and histological data, revealing new information on growth history in a basal hadrosauromorph. Taphonomic characterization of FMNH PR 3847 and associated vertebrate material will allow comparison with other vertebrate localities in the Mussentuchit Member of the Cedar Mountain Formation.

## Introduction

Historically, the early Late Cretaceous (Cenomanian–Santonian [1]) was a poorly understood interval in iguanodontian dinosaur evolution. This was due to the scarcity of fossils with well-constrained ages that postdate the rich Early Cretaceous basal iguanodont record [2,3,4,5,6] and predate the extensive hadrosaurid record from the latter half of the Late Cretaceous (Campanian–Maastrichtian [7,8]). The situation has improved greatly over the last 20 years, with the description of numerous non-hadrosaurid iguanodontians from the Cenomanian–Santonian, including many from Asia (*Nanyangosaurus* [9,10], *Shuangmiaosaurus* [11,12], *Levnesovia* [13], *Batyrosaurus* [14], *Yunganglong* [15], and *Zhanghenglong* [16]).

The North American record of Cenomanian–Santonian iguanodontians also is growing rapidly. Recent discoveries include *Eolambia* from the lower Cenomanian Mussentuchit Member of the Cedar Mountain Formation of Utah [17,18,19,20], *Protohadros* from the middle Cenomanian Woodbine Formation of Texas [21], *Jeyawati* from the middle Turonian Moreno Hill Formation of New Mexico [22], and *Huehuecanauhtlus* from an unnamed Santonian unit in Michoacán, Mexico [23]. Of these, *Eolambia* is by far the most completely known, with two large partial skulls (holotype CEUM 9758 and paratype CEUM 5212 [17,20]) and extensive juvenile material, with nearly every skeletal element represented by multiple examples in the CEUM, OMNH [18,20], and FMNH collections.

*Eolambia* is also the best-known genus of the Cedar Mountain Formation’s burgeoning basal iguanodont record, which spans the lower Barremian through the lower Cenomanian [24] and also includes *Iguanacolossus* [4], *Hippodraco* [4], *Planicoxa* [25,26], and *Cedrorestes* [4,27]. *Eolambia* is also the most abundantly preserved taxon found in the Mussentuchit Member, which also has produced microvertebrate remains of tyrannosauroid, dromaeosaurid, sauropod, ceratopsian, and more basal ornithopod dinosaurs [28], as well as the holotype partial skeleton of the nodosaurid *Animantarx* [29]. A recent intensive effort by the Field Museum of Natural History and the North Carolina Museum of Natural Sciences to collect macrovertebrate remains from the Mussentuchit Member has added greatly to the dinosaur diversity of this assemblage, now known to include the neovenatorid *Siats* [30], a new giant oviraptorosaur [31], and two new ornithopods [32].

Here we describe the anatomy and taphonomy of a new associated partial skeleton of *Eolambia*. This new specimen, FMNH PR 3847, includes the largest and most complete associated pelvic girdle of *Eolambia*, with both ilia, both pubes, and most of the right ischium preserved. The new skeleton supplements previous anatomical descriptions [17,18,20], augments the diagnosis of the taxon, and provides new data with which to test the phylogenetic relationships of *Eolambia*.

## Materials and methods

The specimen of *Eolambia* described in this paper was discovered in 2008 by Akiko Shinya, and excavated in 2009 and 2010 by crews from the Field Museum with excavation permits from the Utah Geological Survey (permit #09–383). The site was mapped using a 1 m by 1 m grid and 167 numbered elements were collected from the quarry. The *Eolambia* bones were disarticulated but associated and in close proximity to each other. The hip elements were concentrated in the quarry and collected in two adjacent plaster jackets. The specimen is accessioned in the Geology collections of the Field Museum as FMNH PR 3847, and was examined, photographed, and measured at that institution.

### Histological analysis

Histological age determination on hadrosaurids is typically performed on limb bones [33,34,35,36] as these appear to preserve the largest number of growth markers in specimens for which multiple elements have been examined [33]. However, as no limb bones were preserved with FMNH PR 3847, we selected a rib for sectioning because ribs have been shown to preserve a good growth record in sauropod [37] and theropod dinosaurs [38].

A small section (~1 cm) was taken from an incomplete caudal dorsal rib from the left side, identified as such by having a tuberculum that is developed as a small saddle shaped depression rather than projecting from the rib shaft and by the nearly continuous arc between the capitulum and rib shaft. The section was taken from the proximal end of the shaft approximately 8 cm below the tuberculum, as Waskow and Sander [37] found this region to preserve more growth markers than more distal rib sections. After cutting, the section was embedded in polyester resin for thin sectioning. The section was adhered to Plexiglass slides using epoxy resin after which thin sections were cut at a thickness of ~0.5 mm. All cutting was carried out on a Buehler Isomet 4000 Linear Precision Saw. Sections were then ground and polished on a Buehler Ecomet 5 using 600/1200 gauge grit paper until a desired thickness was achieved allowing for microscopic examination and photography. The sections were finally buffed using 0.3μm Alumina Microspolish II (Buehler, IL) after the desired thickness was achieved.

The sections were examined and photographed at 4X magnification using an Olympus Bx 60 microscope mounted with an Olympus DP25 digital camera. Images were captured under regular and polarized light using CellSans software run on a Dell Dimension 8400 Pentium IV desktop computer running the Microsoft XP operating system. Individual photos were stitched together in the PT Gui photo stitching software (New House Internet Services BV, Rotterdam) ver. 10.0.15 on an HP 7500 terminal running Windows 7. Lens settings were set to rectilinear lens with a near infinite focal length (100000 mm) as we assumed minimal to no parallax in the micrographs. The stitched panorama and individual polarized micrographs were opened in Adobe Photoshop CS 5 software for cropping and labeling. The polarized light micrograph was sharpened using the sharpening filter to overcome slight differences in focus due to uneven thickness between the middle and peripheral parts of the thin section.

### Phylogenetic analysis

Our phylogenetic analysis is a derivation of the matrix of McDonald et al. [4,39], which was later modified by McDonald [40] and McDonald et al. [41]. The matrix also was used and modified by Barrett et al. [42], Escaso et al. [43], Zheng et al. [44], Shibata and Azuma [45], Gasca et al. [46], and Verdú et al. [47].

We have extensively revised the data matrix. In addition to new anatomical data on *Eolambia caroljonesa* provided by FMNH PR 3847, we have added recently named taxa, consolidated and updated several OTUs based upon recent taxonomic revisions and anatomical descriptions, and excluded several poorly known, equivocal, or dubious OTUs. Additionally, we added new characters and consolidated or otherwise rephrased other characters. The final data matrix includes 47 taxa and 135 characters. *Camptosaurus dispar* was designated as the outgroup. The components of the revised analysis are available as Supporting Information files (S1 and S2 Spreadsheets, S1 Document and S1 File).

The phylogenetic analysis was carried out using a “traditional search” with the tree bisection reconnection algorithm in TNT [48]. The starting trees were Wagner trees with a random seed of 1. We used 7,000 replicates with 10 trees saved per replication. All characters were equally weighted and treated as unordered. The search examined 4,359,364,145 rearrangements and produced 2,060 most parsimonious trees, each with 398 steps. Consensus trees were calculated in TNT.

## Results

### Systematic paleontology

Dinosauria Owen, 1842 [49]

Ornithischia Seeley, 1887 [50]

Ornithopoda Marsh, 1881 [51]

Iguanodontia Dollo, 1888 [52] *sensu* Sereno, 2005 [53]

Ankylopollexia Sereno, 1986 [54] *sensu* Sereno, 2005 [53]

Styracosterna Sereno, 1986 [54] *sensu* Sereno, 2005 [53]

Hadrosauriformes Sereno, 1997 [55] *sensu* Sereno, 1998 [56]

Hadrosauroidea Cope, 1870 [57] *sensu* Sereno, 2005 [53]

Hadrosauromorpha Norman, 2014 [58]

*Eolambia caroljonesa* Kirkland, 1998 [17]

#### Holotype (after Kirkland, 1998 [17])

CEUM 9758, partial adult skull and associated postcranium, from CEUM Locality 42em366v, east of Castle Dale, Utah.

#### Paratypes (after Kirkland, 1998 [17] and Head, 2001 [18])

CEUM 5212, partial adult skull, from CEUM Locality 42em369v; two partial juvenile skeletons from OMNH Locality v237; partial juvenile skeleton from OMNH Locality v824; OMNH 27749, sacrum and ischium, from OMNH Locality v696; OMNH 24389, isolated left ischium, from OMNH Locality v214; OMNH 32812, scapula and two caudal vertebrae, from OMNH Locality v866.

#### Referred Material (modified from McDonald et al., 2012 [20])

**Fig 1:**
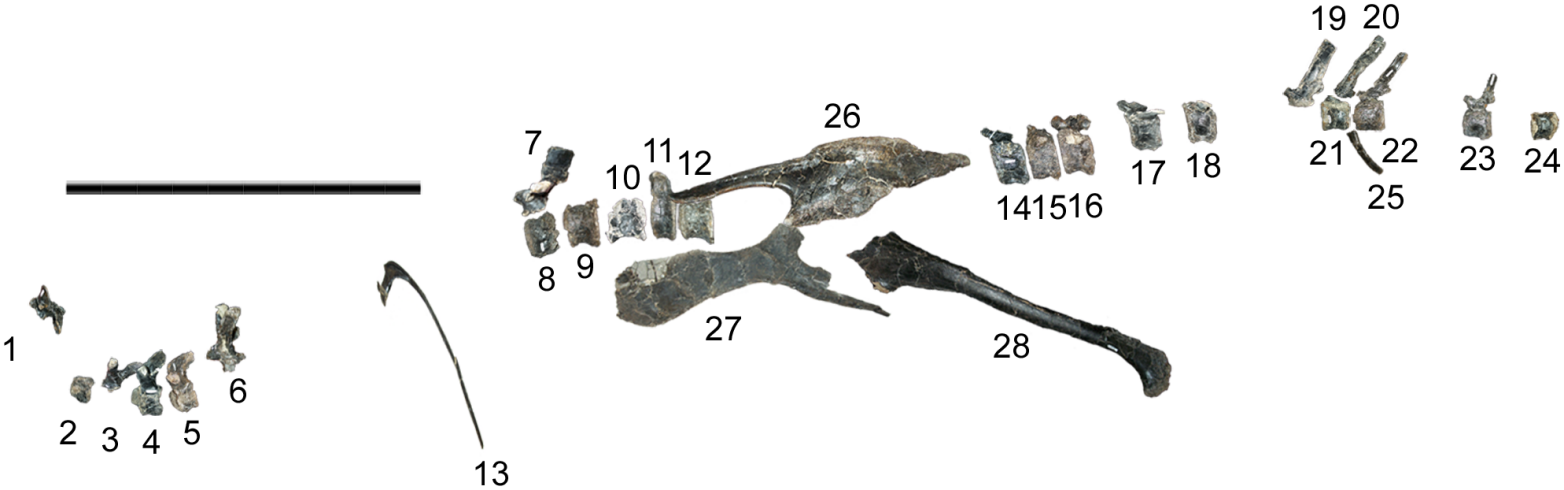
Preserved elements of FMNH PR 3847. Bones are arranged in an approximation of life position in left lateral view, using a skeletal reconstruction of *Mantellisaurus atherfieldensis* [62] as a template. Numbers correspond to table of measurements (S1 Table). Caudal cervical vertebra (4), caudal dorsal neural arch (7), right dorsal rib (13), two proximal caudal centra (17 and 18), left pubis (27, medial view), and right ischium (28) are reversed. Sacral ribs and more recently prepared dorsal vertebrae are not shown. Scale bar equals 1 meter.

CEUM 8786, isolated left femur from the same locality as CEUM 9758 (42em366v), approximately 100 meters southwest of the holotype quarry at the same stratigraphic level. Disarticulated juvenile cranial and postcranial material (CEUM collection, MNI = 12) from the *Eolambia* #2 quarry (CEUM locality 42em432v) in Mussentuchit Wash, south of Emery, Utah. Disarticulated juvenile cranial and postcranial material (CEUM collection, MNI = 4) from the Willow Springs 8 quarry (CEUM locality 42em576v) in Mussentuchit Wash, south of Emery, Utah. FMNH PR 3847, partial postcranial skeleton consisting of cervical vertebrae, dorsal vertebrae and ribs, sacral ribs, caudal vertebrae and chevrons, the left and right ilia, left and right pubes, and right ischium (Fig 1), from Akiko’s Site quarry (FMNH locality UT080821-1), south of Emery, Utah. Disarticulated juvenile cranial and postcranial material (>400 bones in FMNH collection, MNI = 4) from the Triple Peak quarry (FMNH locality UT130904-2) on south side of Mussentuchit Wash, south of Emery, Utah. Specific coordinates for the latter two sites are on file at FMNH.

#### Specific Diagnosis (as for genus by monotypy; modified from McDonald et al., 2012 [20])

Character based solely upon skeletally immature specimens is marked with an asterisk. Basal hadrosauroid diagnosed by the following unique combination of characters: rostral ramus of dentary deepens in lateral view (also in *Ouranosaurus* [59], *Protohadros* [21], and *Bactrosaurus* (AMNH 6553)); dorsal end of coronoid process expanded along only rostral margin* (also in *Fukuisaurus* [60], *Iguanodon* (MIWG 1997.55) [61], *Mantellisaurus* (NHMUK R5764) [62], *Bolong* [63], *Penelopognathus* [64], *Protohadros* [21], and *Jeyawati* [22], but different from *Proa* [41] and *Probactrosaurus* [65], in which it is expanded along both the rostral and caudal margins); ventral margin of maxillary tooth row concave in lateral view (also in *Iguanacolossus* [4], *Dakotadon* (SDSM 8656) [66], *Fukuisaurus* [60], *Iguanodon* [61], *Mantellisaurus* (NHMUK R5764) [62], *Proa* [41], *Ouranosaurus* [59], *Altirhinus* [67], *Equijubus* [68], *Probactrosaurus* [65], *Shuangmiaosaurus* [11], and *Zhanghenglong* [16], but different from *Bolong* [63], *Xuwulong* [69], *Siamodon* [70], *Protohadros* [21], and *Jeyawati* [22], in which it is straight); dentary teeth with a primary ridge and single mesial accessory ridge (present in holotype CEUM 9758; also in *Protohadros* [21], *Levnesovia* [13] *Tethyshadros* [71], *Huehuecanauhtlus* [23], *Zhanghenglong* [16], and some specimens of *Bactrosaurus* [72]); straight shaft of ischium (also in *Uteodon* [26,73], *Mantellisaurus* (IRSNB 1551, NHMUK R3741), *Bolong* [63], *Altirhinus* [67], *Bactrosaurus* [72], *Gilmoreosaurus* [74], and *Yunganglong* [15]); straight distal half of femoral shaft (also in *Hypselospinus* (NHMUK R1629 [58]), *Iguanodon* [61], *Proa* [41], *Ouranosaurus* [59], *Gongpoquansaurus* [75,76], *Nanyangosaurus* [9], *Bactrosaurus* [72], *Gilmoreosaurus* [74], *Tanius* [77], and *Tethyshadros* [71]); dorsally-directed flange along the dorsal margin of the ilium, extending from above the acetabulum to the postacetabular process (also in hadrosauroid material from the Lewisville Member of the Woodbine Formation of Texas [78]).

#### Distribution and Horizon

All specimens of *Eolambia caroljonesa* have been found in Emery County, Utah, in the Mussentuchit Member, Cedar Mountain Formation (lower Cenomanian) [17,18,19,20]. More precise locality data are on file at CEUM, OMNH, and FMNH.

### Description of FMNH PR 3847

Measurements of FMNH PR 3847 are given in the online Supporting Information (S1 Table). The approximate positions of the disarticulated but associated cervical, dorsal, and caudal vertebrae of FMNH PR 3847 were derived through comparison with iguanodontians for which complete, articulated vertebral columns are known, especially *Iguanodon bernissartensis* [61] and *Mantellisaurus atherfieldensis* [62]. Comparison also was made with the disarticulated *Eolambia* bonebed material described by McDonald et al. [20].

#### Cervical vertebrae

FMNH PR 3847 includes the neural arch of a middle cervical vertebra, the complete right half of a caudal cervical vertebra, the left half of the neural arch of another caudal cervical vertebra, and an eroded opisthocoelous centrum of uncertain position in the cervical column (Fig 1).

**Fig 2:**
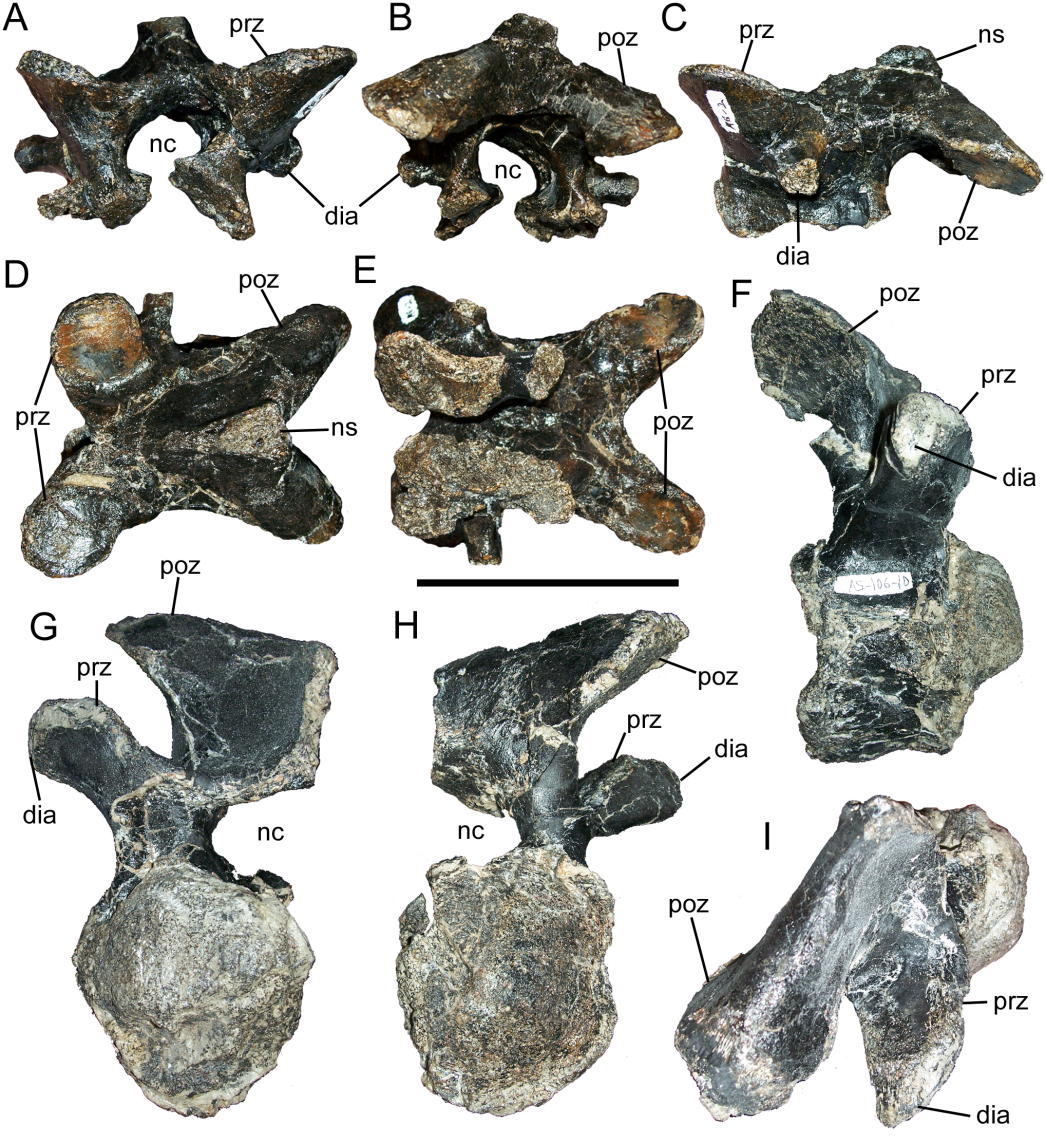
Cervical vertebrae of FMNH PR 3847. Middle cervical neural arch in (A) cranial, (B) caudal, (C) left lateral, (D) dorsal, and (E) ventral views. Right half of caudal cervical vertebra in (F) right lateral, (G) cranial, (H) caudal, and (I) dorsal views. *Abbreviations*: *dia*, diapophysis; *nc*, neural canal; *ns*, neural spine; *poz*, postzygapophysis; *prz*, prezygapophysis. Scale bar equals 10 cm.

The middle cervical neural arch is complete except for the neural spine. The neural canal is elliptical, with the long axis oriented mediolaterally, although there appears to be some distortion (Fig 2A and 2B). The prezygapophyses are pedestals that project dorsolaterally from the cranial margin of the neural arch, terminating in subcircular, dorsomedially-facing articular surfaces (Fig 2A, 2C and 2D). The blunt, rod-like diapophyses are caudoventral to the prezygapophyses, on the lateral surfaces of the neural arch, and project laterally (Fig 2A–2C). Caudal to the prezygapophyses, the dorsal surface of the neural arch slopes gently dorsally towards the subtriangular base of the neural spine (Fig 2C and 2D). Caudal to the base of the neural spine, the neural arch splits into two elongate postzygapophyses that project caudolaterally and curve slightly ventrally along their lengths; each postzygapophysis terminates in an ovoid, ventrolaterally-facing articular surface (Fig 2B–2E).

The centrum of the caudal cervical vertebra is strongly opisthocoelous, with the cranial surface forming a hemispherical bulge and the caudal surface a deep concavity (Fig 2F–2H); this morphology is present in many styracosternans, including *Hippodraco* [4], *Lurdusaurus* [79], *Lanzhousaurus* [80], *Barilium* [81], *Hypselospinus* [58], *Iguanodon* [61], *Mantellisaurus* [62], *Ouranosaurus* [59], *Jinzhousaurus* [82], *Bolong* [63], *Equijubus* [68], *Probactrosaurus* [65], *Jeyawati* [22], *Bactrosaurus* [72], *Gilmoreosaurus* [74], *Tanius* [77], *Huehuecanauhtlus* [23], *Yunganglong* [15], *Claosaurus* [83], and hadrosaurids [7]. The right lateral surface of the centrum is damaged and the left is missing, precluding description of the parapophyses. In contrast, the right side of the neural arch is complete and well preserved. As in the middle cervical neural arch described above, the neural canal appears to have been elliptical (Fig 2G and 2H). Rather than forming a distinct pedestal as in the middle cervical neural arch, the prezygapophysis of the caudal cervical is a cranially-convex ledge on the craniodorsal surface of a large transverse process (Fig 2F–2I). The rounded diapophysis is at the end of the transverse process (Fig 2F–2I). The postzygapophysis of the caudal cervical projects caudolaterally and terminates in a ventrolaterally-facing articular surface, similar to the postzygapophyses of the middle cervical neural arch (Fig 2F–2I). However, the postzygapophysis of the caudal cervical lacks the slight ventral curvature of those of the middle cervical, instead projecting dorsally for its entire length. Furthermore, although the left side of the neural arch is missing, the postzygapophyses of the caudal cervical clearly would have been more divergent, with a greater angle between them in dorsal view, than those of the middle cervical neural arch.

#### Dorsal vertebrae and ribs

FMNH PR 3847 includes the nearly intact first dorsal vertebra, two nearly complete cranial dorsal vertebrae missing only the distal ends of their neural spines, the complete neural arches of two additional cranial dorsal vertebrae, the complete neural arch of a caudal dorsal vertebra, and the centra of five caudal dorsal vertebrae (Fig 1). The specimen also includes 11 dorsal ribs in various states of preservation.

**Fig 3:**
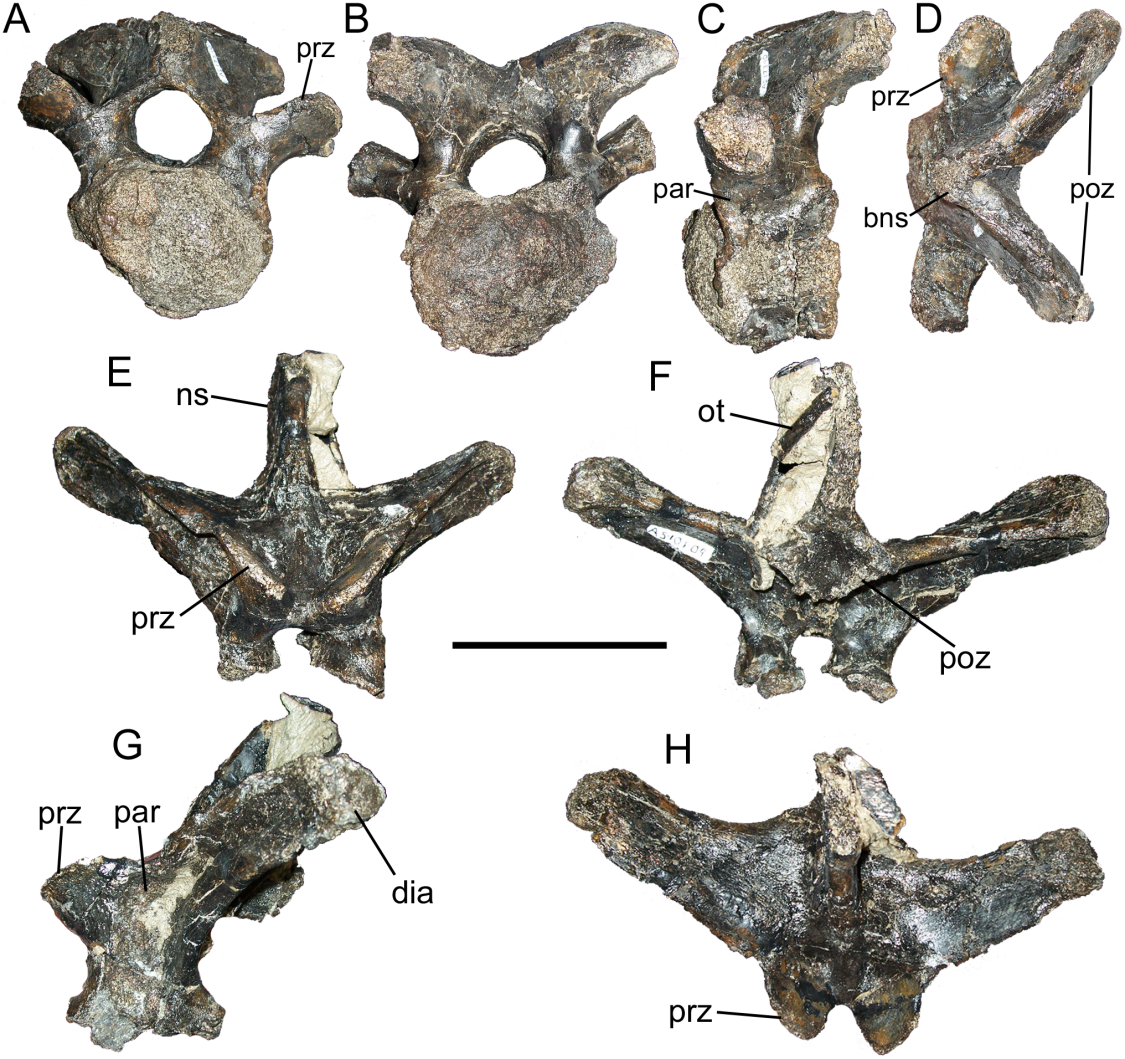
Cranial dorsal vertebrae of FMNH PR 3847. D1 in (A) cranial, (B) caudal, (C) left lateral, and (D) dorsal views. Neural arch of D3 or D4 in (E) cranial, (F) caudal, (G) left lateral, and (H) dorsal views. *Abbreviations*: *bns*, base of neural spine; *dia*, diapophysis; *ns*, neural spine; *ot*, ossified tendon; *par*, parapophysis; *poz*, postzygapophysis; *prz*, prezygapophysis. Scale bar equals 10 cm.

The first dorsal vertebra (D1) was identified as such based upon its combination of an opisthocoelous centrum; parapophyses located on the lateral surfaces of the neural arch, rather than on the centrum; and small neural spine, as indicated by the size of the broken base (Fig 3A–3D). In this combination of features, the D1 of FMNH PR 3847 compares closely with those of *Iguanodon* [61] and *Mantellisaurus* [62], for which articulated vertebral series are known. D1 is similar to the caudal cervical vertebra described above in the aforementioned opisthocoelous centrum, as well as prezygapophyses that form ledges on the dorsal surfaces of the large, laterally-projecting transverse processes and elongate, caudolaterally-directed, and widely divergent postzygapophyses (Fig 3A–3D).

The neural arch of another cranial dorsal vertebra probably represents either D3 or D4 based upon comparison with the dorsal series of *Iguanodon* [61] and *Mantellisaurus* [62]. The prezygapophyses are oblong, tab-like, and project cranially, with the articular surfaces steeply inclined ventromedially (Fig 3E, 3G and 3H). Each prezygapophysis is bordered caudomedially by a small but well-defined depression, and a low ridge separates these depressions along the midline. This ridge is better developed in more caudally positioned vertebrae, and is observed in the mid-dorsal and caudal dorsal vertebrae of *Edmontosaurus* [84]. The parapophyses are shallow, rugose, and roughly elliptical depressions on the lateral surfaces of the neural arch, immediately caudal to the prezygapophyses and cranial to the bases of the transverse processes (Fig 3G). They extend ventrally nearly to the neurocentral suture as in the cranial dorsals of *Edmontosaurus* [84], and unlike the other preserved dorsal vertebrae that all have parapophyses situated farther from the neurocentral suture. The transverse processes sweep dorsolaterally in cranial and caudal views, and caudolaterally in dorsal view (Fig 3E–3H), terminating in rounded, rugose diapophyses (Fig 3G). Each transverse process is composed of three laminae, one extending from the prezygapophysis to the diapophysis (Fig 3E), another from the postzygapophysis to the diapophysis (Fig 3F), and a third from near the neurocentral suture to the diapophysis (Fig 3G); the dorsal surfaces of the transverse processes are flat (Fig 3H). The neural spine arises between the transverse processes and, though incomplete along its dorsal and caudal margins, appears to have been subrectangular (Fig 3E–3H). The postzygapophyses are ventral to the caudal margin of the neural spine and form a pair of ventrolaterally-facing ledges with a quadrangular depression between them (Fig 3F). A rod-like fragment of ossified tendon is adhered by matrix to the left lateral surface of the neural spine (Fig 3F).

A nearly complete vertebra with the centrum and arch in articulation likely represents the next element (D5?) following the arch described above. The centrum has a flat cranial intercentral articulation and a faintly concave caudal one, and lacks a ventral keel. The neural canal is elliptical cranially but circular in caudal view. The parapophyses are elongate with a dorsoventral long axis, but do not reach the neurocentral suture ventrally. The prezygapophyses are situated close to the midline and separated by a distinct ridge below the base of the neural spine. The neural spine has a broad base in lateral view, and is inclined caudally. Although it is missing its distal section, the spine is taller than its corresponding centrum. The transverse processes are less backswept than in the preceding neural arch, and separated from the postzygapophyses by a sharp but shallow notch in dorsal view. A thick, tear-drop shaped septum projects ventrally from the midline where the postzygapophyses meet. A shallow depression occupies the base of the neural spine between the postzygapophyses in caudal view.

Another vertebra bears a neural arch that is similar in anatomy to the preceding element, but has a wider base to its neural spine, a circular neural canal opening in cranial view and parapophyses even farther removed from the neurocentral suture. The transverse processes are shorter and wider in dorsal view, and are separated from the postzygapophyses by a distinct notch. The midline septum projecting ventrally below the postzygapophyses is well developed and tapers caudally in ventral view. These traits suggest a more caudal position for this vertebra, perhaps as D6 or D7. A centrum was found disarticulated from, but adjacent to and touching this arch. The centrum has a slightly convex cranial intercentral articulation and a weak ventral keel typical of more cranial dorsal vertebrae [84], and may not belong with the arch despite their close proximity.

A more caudally positioned neural arch exhibits parapophyses that do not extend below the level of the prezygapophyses, which in turn have less inclined articulations than those of the preceding vertebrae. The transverse processes are slender and less backswept than in more cranial elements and are not separated caudally from the poszygapophyses by a notch. The postzygapophyseal septum is robust in ventral view. The neural spine is caudally inclined and its base overhangs the arch peduncles by a wide margin. Comparison to the vertebral column of *Iguanodon* [61] suggests a position as the 10^th^ dorsal vertebra.

An isolated dorsal centrum with a narrow neural canal and a well developed keel appears to be from a similar position along the vertebral column, but taphonomic distortion of both the centrum and arch render a fit between the two uncertain. The cranial intercentral articulation is taller than wide and flat, whereas the caudal face is markedly wider as in the caudal dorsal vertebrae of *Iguanodon* [61].

**Fig 4:**
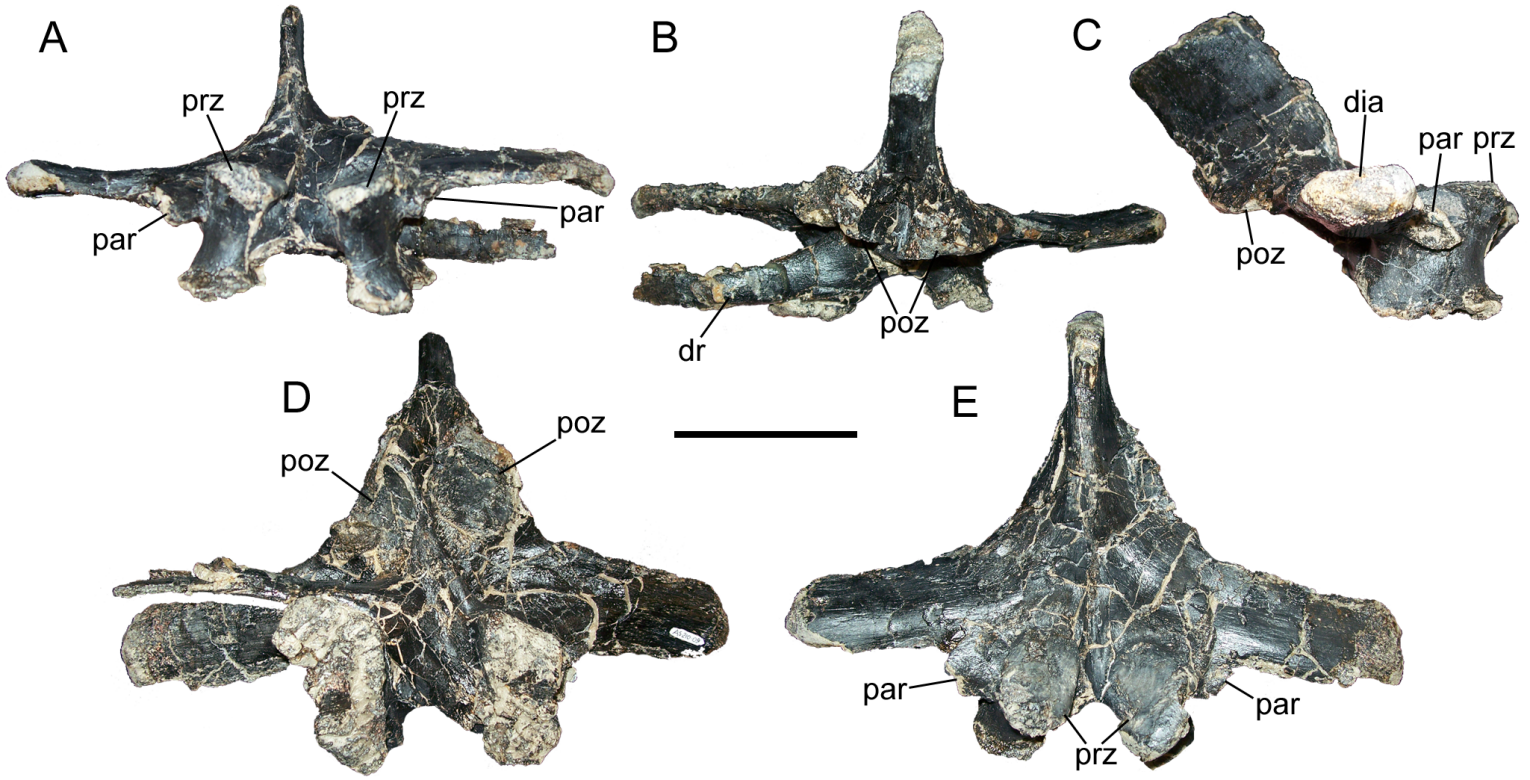
Caudal dorsal neural arch of FMNH PR 3847. Neural arch in (A) cranial, (B) caudal, (C) right lateral, (D) ventral, and (E) dorsal views. *Abbreviations*: *dia*, diapophysis; *dr*, dorsal rib fragment; *par*, parapophysis; *poz*, postzygapophysis; *prz*, prezygapophysis. Scale bar equals 10 cm.

Two neural arches of caudal dorsal vertebrae probably represent either D12 and D13, or D13 and D14, bearing a close resemblance to those vertebrae of *Iguanodon* [61] and *Mantellisaurus* [62] in the horizontal, laterally-directed transverse processes and positions of the parapophyses at the cranial margins of the bases of the transverse processes (Fig 4A–4E). The parapophyses themselves are laterally-protruding, subcircular structures, each forming a cup-like depression (Fig 4A, 4C and 4E). Both differ from the preceding dorsal vertebrae in that the articular surfaces of the prezygapophyses are nearly horizontal (Fig 4A), the transverse processes are simple flat structures lacking distinct laminae (Fig 4A–4E), the neural spines are displaced caudally beyond the arch peduncles (Fig 4C), and the postzygapophyses face ventrally rather than ventrolaterally (Fig 4B and 4D). There is a short section of a dorsal rib attached to the neural arch near the left neurocentral suture of the more caudal of the two arches (Fig 4B), identified as such by its smaller prezygapophyses.

**Fig 5:**
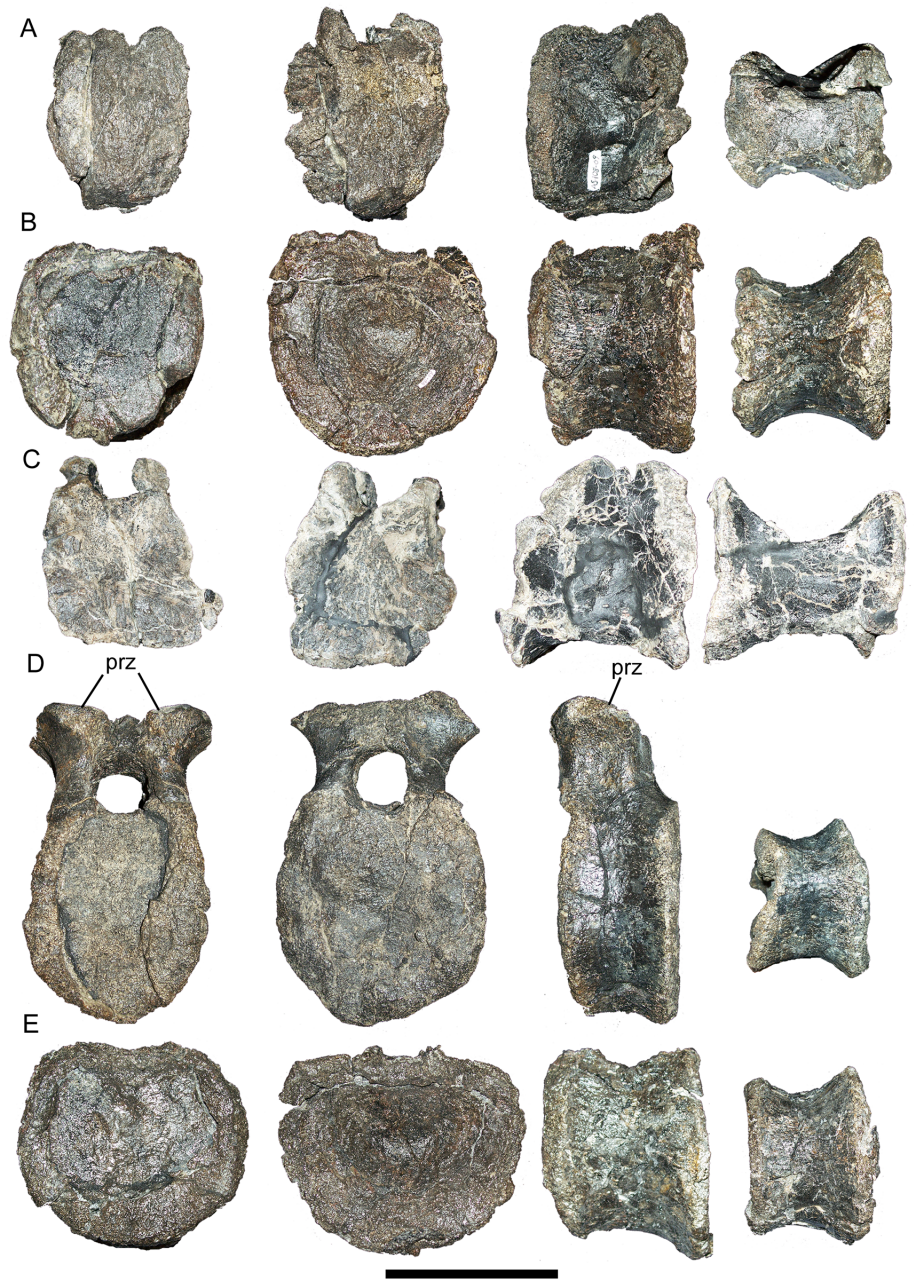
Caudal dorsal centra of FMNH PR 3847. Centra of D12 (A), D13 (B), D14 (C), D15 (D), and D16 (E) in cranial, caudal, left lateral, and ventral views (left to right). *Abbreviations*: *prz*, prezygapophyses. Scale bar equals 10 cm.

Based upon their shapes and comparisons with *Iguanodon* [61] and *Mantellisaurus* [62], the five dorsal centra might represent D12–D16. One of these appears to fit reasonably well with the arch we tentatively identify as D12. All five centra are amphiplatyan with subcircular articular faces (Fig 5A–5E). In all five centra, the caudal articular face is transversely wider than the cranial face, making the centra spool-shaped in lateral and ventral views (Fig 5A–5E). The ventral margins of the centra are concave in lateral view and there is an offset between the cranial and caudal faces. The centrum identified as D15 retains a small portion of the neural arch; the only recognizable features are the prezygapophyses, which closely resemble those of the caudal dorsal neural arch (D12 or D13) described above, except that they are craniocaudally shorter and face dorsally (Fig 5D).

**Fig 6:**
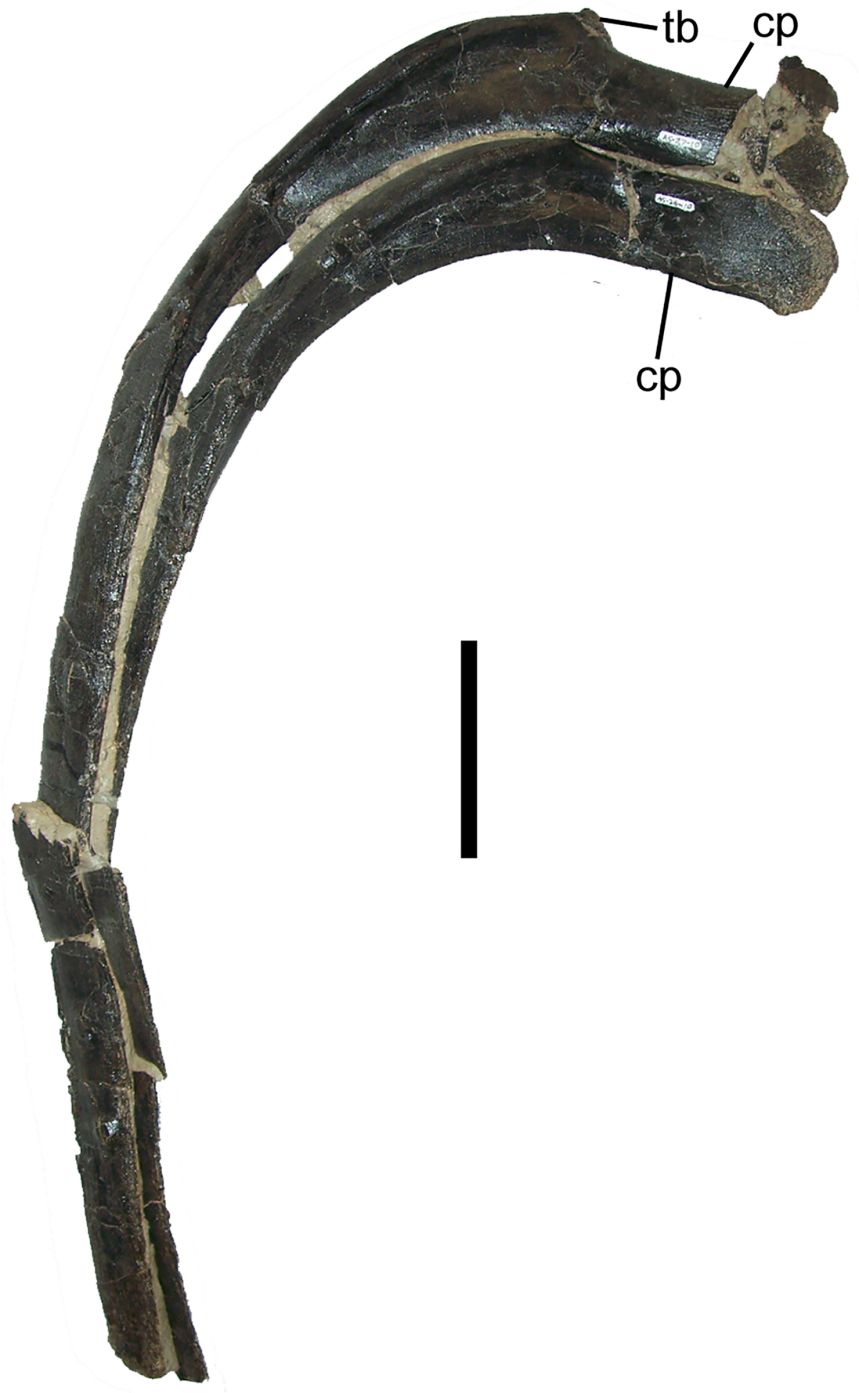
Dorsal ribs of FMNH PR 3847. Two right dorsal ribs in cranial view. *Abbreviations*: *cp*, capitulum; *tb*, tuberculum. Scale bar equals 10 cm.

The proximal ends of the dorsal ribs bifurcate into the ventral capitulum and the dorsal tuberculum. The subrectangular capitulum is craniocaudally compressed; the proximal end of the capitulum, where it would have articulated with the parapophysis of a dorsal vertebra, is rounded and rugose (Fig 6). The subtriangular tuberculum is located at the base of the capitulum and projects dorsally (Fig 6). The dorsal ribs of FMNH PR 3847 closely resemble those of juvenile *Eolambia* [20], and those of other iguanodontians, such as *Iguanodon* [61], *Mantellisaurus* [62], and *Jinzhousaurus* [82].

#### Sacral ribs

**Fig 7:**
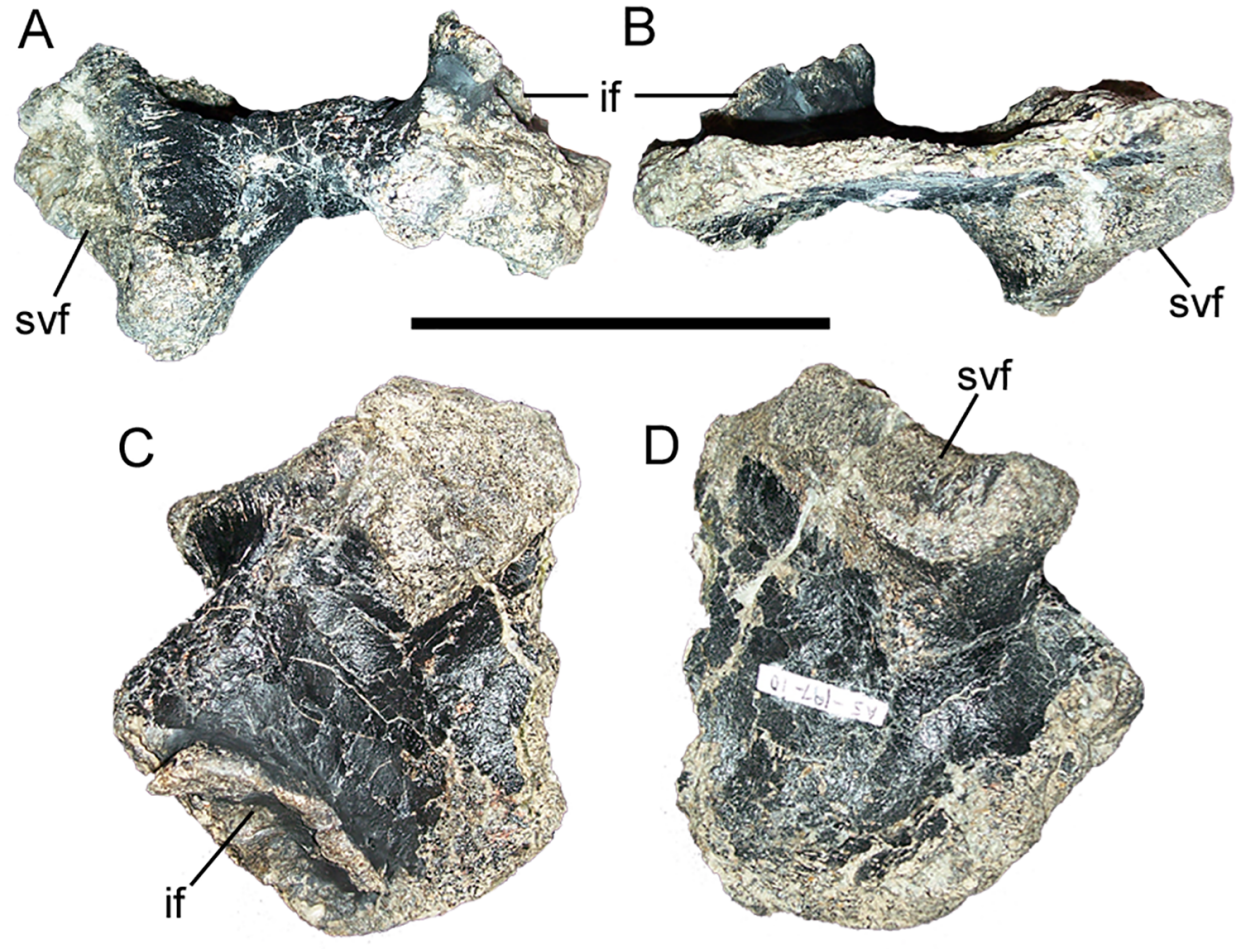
Sacral rib of FMNH PR 3847. Left fifth sacral rib in (A) cranial, (B) caudal, (C) dorsal, and (D) ventral views. *Abbreviations*: *if*, facet for articulation with ilium; *svf*, facet for articulation with sacral vertebrae. Scale bar equals 10 cm.

FMNH PR 3847 includes four disarticulated sacral ribs in various states of preservation. The most complete is tentatively identified as the fifth sacral rib from the left side, based upon comparisons with the articulated sacra of *Iguanodon* [61], *Mantellisaurus* [62], and *Bactrosaurus* [72]. The medial surface of the rib forms a rugose, ventrally-expanded facet for articulation with the fifth and sixth sacral vertebrae (Fig 7A and 7B). Lateral to the articular surface, the rib expands craniocaudally (Fig 7C and 7D), yet becomes markedly thinner dorsoventrally, especially along the caudal margin (Fig 7A and 7B). The lateral margin of the rib is dorsally expanded and rugose, forming a facet at which the rib would articulate with the corresponding facet on the medial surface of the ilium (Fig 7A and 7B).

#### Caudal vertebrae and chevrons

FMNH PR 3847 includes the centra of five proximal caudal vertebrae, the neural spines of two proximal caudal vertebrae, two nearly complete middle caudal vertebrae, the centra of two additional middle caudal vertebrae, and two complete chevrons (Fig 1).

**Fig 8:**
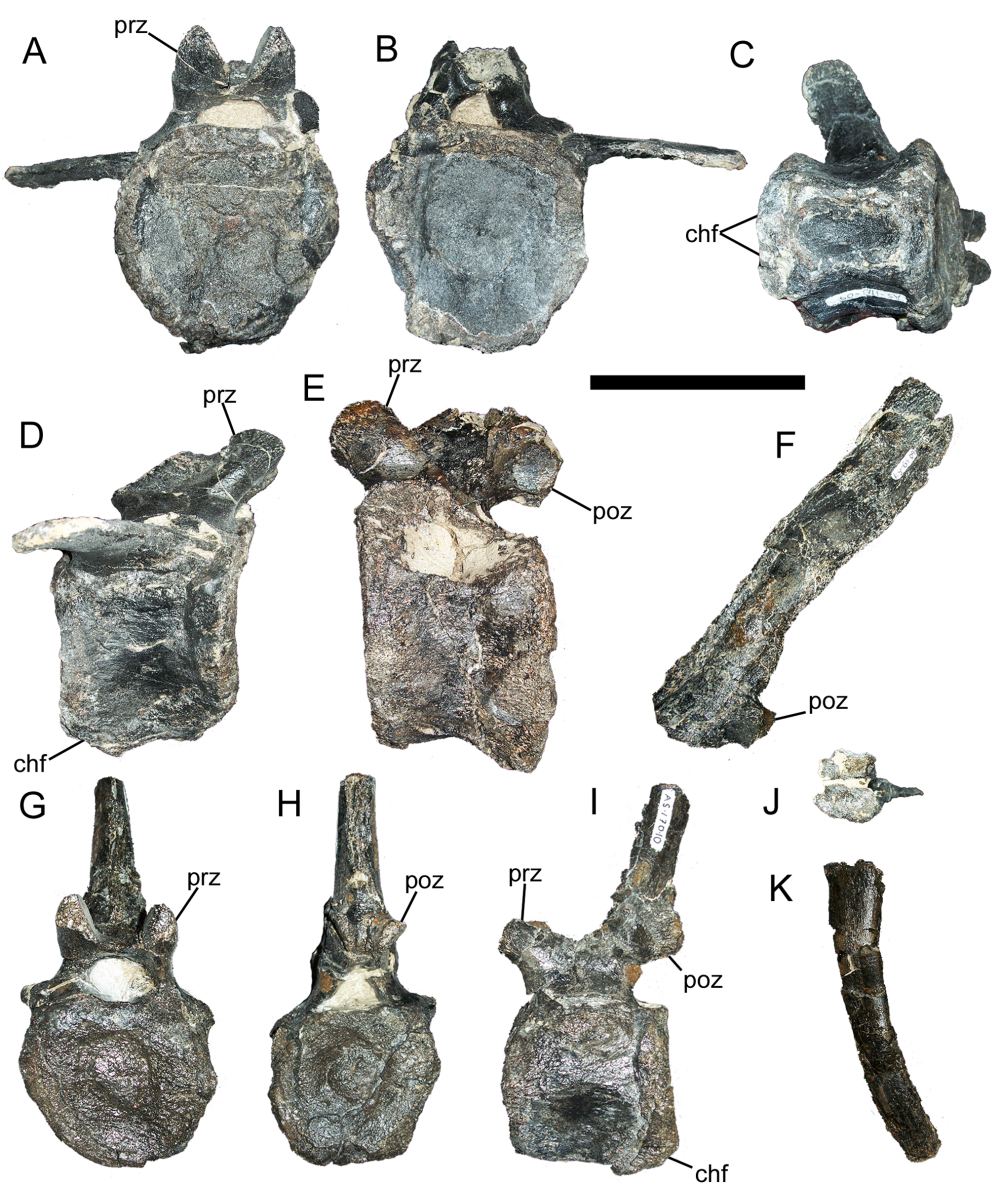
Caudal vertebrae and chevron of FMNH PR 3847. Proximal caudal centrum in (A) cranial, (B) caudal, (C) ventral, and (D) right lateral views. Proximal caudal centrum in (E) left lateral view. Proximal caudal neural spine in (F) left lateral view. Middle caudal vertebra in (G) cranial, (H) caudal, and (I) left lateral views. Proximal chevron in (J) proximal and (K) left lateral views. *Abbreviations*: *chf*, chevron facet; *poz*, postzygapophyses; *prz*, prezygapophyses. Scale bar equals 10 cm.

The cranial and caudal articular faces of a proximal caudal centrum are subcircular and gently concave (Fig 8A and 8B). The ventral surface of the centrum bears two craniocaudally-directed ridges that are laterally offset from the midline of the centrum (Fig 8C); caudally, these ridges terminate in a pair of prominent facets for the articulation of a proximal chevron (Fig 8C and 8D). Between the ridges is a deep elliptical depression. The caudal ribs are dorsoventrally compressed and project caudolaterally from the dorsolateral surface of the centrum (Fig 8A–8D). The prezygapophyses project craniodorsally and face dorsomedially (Fig 8A, 8D and 8E). The postzygapophyses are located on the caudal margin of the base of the neural spine and face ventrolaterally (Fig 8E and 8F). The neural spine is straight and inclined caudally, with a rectangular distal end (Fig 8F).

The middle caudal vertebrae are broadly similar to the proximal caudals, with slightly concave articular faces and prominent chevron facets on the centra, dorsomedially-facing prezygapophyses, and ventrolaterally-facing postzygapophyses on the base of the neural spine (Fig 8G–8I). However, the middle caudal vertebrae differ from the more proximal members of the series in having hexagonal, rather than subcircular, articular faces on the centra; caudal ribs reduced to small, rounded nubs; more elongate prezygapophyses; and neural spines that curve dorsally, with gently concave cranial margins and convex caudal margins (Fig 8G–8I).

The proximal end of the proximal chevron is mediolaterally and craniocaudally expanded into two articulatar facets proximal to the haemal canal (Fig 8J). The distal portion of the chevron curves caudally and is rounded at its distal end (Fig 8K). A second, smaller chevron is similar in its anatomy, but is about two thirds the length of the more proximal element.

#### Ilium

**Fig 9:**
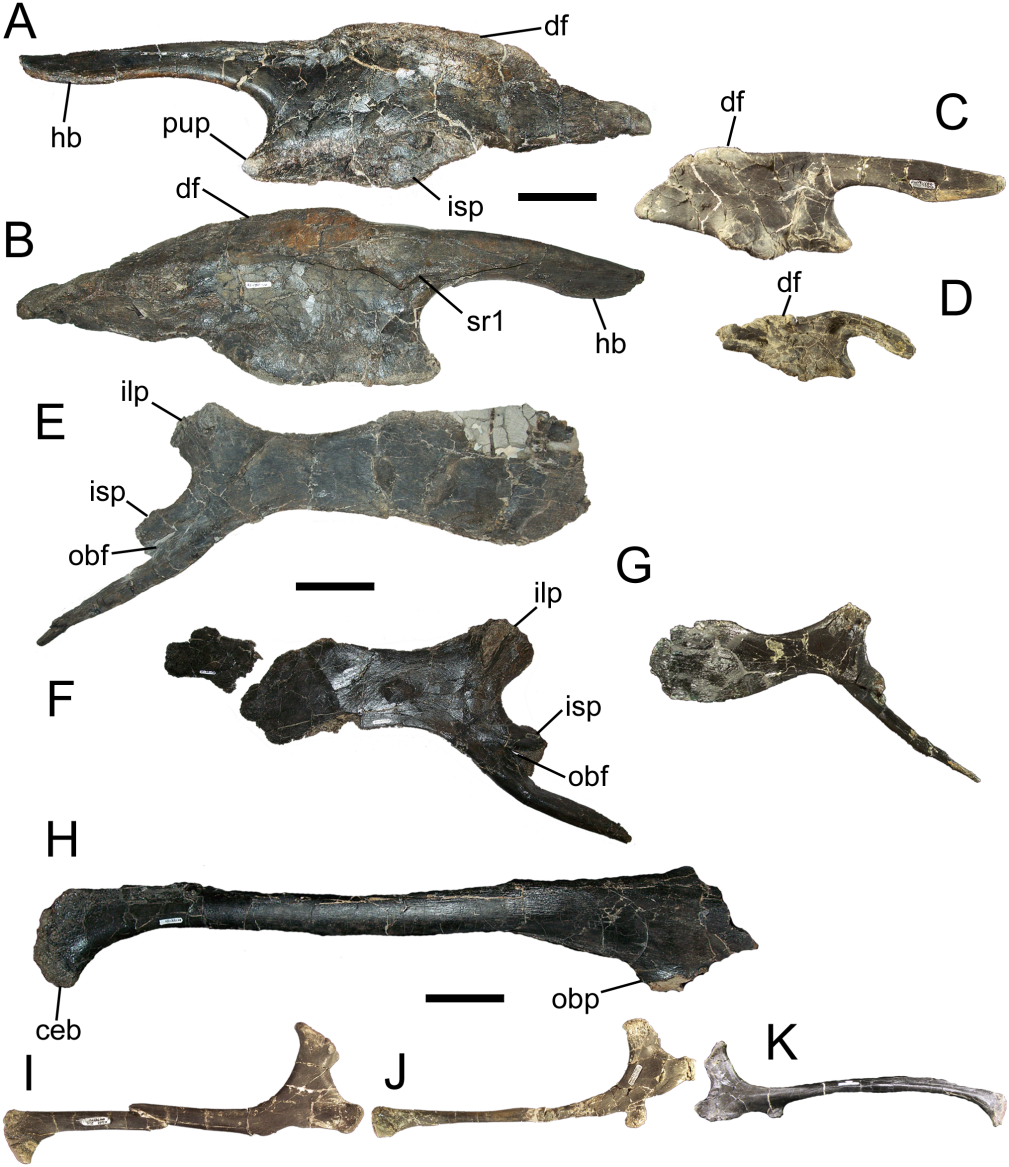
Pelvic elements of *Eolambia*. Left ilium of FMNH PR 3847 (A) lateral and (B) medial views. Right ilium CEUM 52090 in (C) lateral view. Right ilium CEUM 14601 in (D) lateral view. Left (E) and right (F) pubes of FMNH PR 3847 in medial view. Right pubis CEUM 52152 in (G) medial view. Right ischium of FMNH PR 3847 in (H) lateral view. Right ischia CEUM 74572 (I) and CEUM 52941 (J) in lateral view. Left ischium FMNH PR 3847 in (K) lateral view. *Abbreviations*: *ceb*, cranially-expanded boot; *df*, dorsal flange; *hb*, horizontal boot; *ilp*, iliac peduncle; *isp*, ischial peduncle; *obf*, obturator foramen; *obp*, obturator process; *pup*, pubic peduncle; *sr1*, facet for first sacral rib. Scale bars equal 10 cm.

FMNH PR 3847 includes the intact, undistorted left ilium (Fig 1). The right ilium is also preserved, but exhibits a break near the base of the preacetabular process as well as poorer preservation of periosteal surfaces, so our description will focus on the better preserved left element. The preacetabular process projects almost straight cranially, with little change in slope between the dorsal margin of the process and that of the body of the ilium dorsal to the pubic peduncle (Fig 9A and 9B). The distal end of the preacetabular process is ventrally expanded into a horizontal ‘boot’, as in many other styracosternans, including *Iguanacolossus* [4], *Cedrorestes* [27], *Planicoxa* [25], *Osmakasaurus* [26,73], *Barilium* [81], *Iguanodon* [61], *Mantellisaurus* [62], *Ouranosaurus* [59], *Xuwulong* [69], and *Probactrosaurus* [65]. The medial surface of the preacetabular process exhibits a mediolaterally-thickened shelf that becomes dorsoventrally deeper caudally, reaching its greatest depth dorsal to the pubic peduncle. Immediately cranial to this deepest point, the shelf bears a well-demarcated facet for the articulation of the first sacral rib (Fig 9B); the other sacral rib facets are indistinct. The shelf extends farther caudally along the body of the ilium, terminating near the caudal end of the medial surface of the postacetabular process and forming a narrow brevis fossa.

The subtriangular pubic peduncle and oval, ventrolaterally-facing ischial peduncle define a broad, shallow acetabulum (Fig 9A). The dorsal margin of the body of the ilium exhibits a dorsally-directed flange extending from above the approximate midpoint of the acetabulum to a point on the dorsal margin of the postacetabular process caudal to the ischial peduncle (Fig 9A and 9B). A similar flange is also present on ilia of skeletally immature individuals from the *Eolambia* #2 quarry in the CEUM collection, albeit subtler, extending only as far cranially as the ischial peduncle (Fig 9C and 9D). A similar dorsal flange also is present on a hadrosauroid ilium from the Lewisville Member of the Woodbine Formation [78]. The postacetabular process tapers towards its caudal end with no break in the slope of its dorsal margin, as in *Iguanodon* [61], *Bactrosaurus* [72], *Gilmoreosaurus* [74], and *Claosaurus* [83]. The length of the postacetabular process relative to the body of the ilium appears to increase through ontogeny (Fig 9A, 9C and 9D).

#### Pubes

FMNH PR 3847 includes the nearly complete left pubis and the right pubis missing most of the distal end of the cranial pubic process (Fig 1). The distal end of the cranial pubic process is dorsoventrally expanded, as in *Lurdusaurus* [79], *Barilium* [81], *Hypselospinus* [58], *Lanzhousaurus* [80], *Iguanodon* [61], *Mantellisaurus* [62], *Delapparentia* [85], *Ouranosaurus* [59], *Altirhinus* [67], *Bolong* [44], *Xuwulong* [69], *Gongpoquansaurus* [75,76], *Probactrosaurus* [65], *Bactrosaurus* [72], *Gilmoreosaurus* [74], *Levnesovia* [13], *Tethyshadros* [71], *Huehuecanauhtlus* [23], and hadrosaurids [7]. The dorsoventral expansion of the cranial pubic process is asymmetrical, with the ventral margin more expanded than the dorsal, giving the dorsoventral expansion an apparent cranioventral inclination (Fig 9E). The rectangular iliac peduncle projects caudodorsally (Fig 9E and 9F). The ischial peduncle projects caudoventrally and meets a smaller, more distal process on the caudal pubic process to frame the obturator foramen (Fig 9E and 9F). The caudal pubic process is considerably shorter than the ischium and tapers to a point at its distal end. The pubes of FMNH PR 3847 closely resemble a complete juvenile pubis from the Willow Springs 8 quarry in the CEUM collection (Fig 9G), although the expansion of the ventral margin appears to be more pronounced in the larger FMNH PR 3847 (Fig 9E and 9G).

#### Ischium

FMNH PR 3847 includes the shaft of the right ischium (Fig 1). The proximal end is craniocaudally expanded; however, the iliac and pubic peduncles are missing and only the base of the obturator process is present. The shaft is straight, as in the dryosaurid *Valdosaurus* [42], *Uteodon* [26,73], *Mantellisaurus* (IRSNB 1551, NHMUK R3741) [62], *Altirhinus* [67], *Bolong* [63], *Bactrosaurus* [72], *Gilmoreosaurus* [74], and *Yunganglong* [15]. The distal end of the ischium is a cranially-expanded boot (Fig 9H). The preserved portion of the ischium of FMNH PR 3847 is similar in form to smaller ischia from skeletally immature specimens of *Eolambia* in the CEUM collection (Fig 9I and 9J). The FMNH has collected at least three other ischia from skeletally immature individuals, but unlike the ischia of FMNH PR 3847 and the CEUM sample, the shafts of these juvenile ischia curve cranially as in many other iguanodontians (Fig 9K), such as *Camptosaurus* (e.g., USNM 4282, YPM PU14553) [86], *Hypselospinus* (NHMUK R811) [58], *Iguanodon* (e.g., IRSNB 1534) [61], *Ouranosaurus* [59], *Jinzhousaurus* [82], *Xuwulong* [69], *Probactrosaurus* [65], *Nanningosaurus* [87], and *Nanyangosaurus* [9]. These new elements demonstrate that the curvature of the ischial shaft might vary in some iguanodontian species over ontogeny.

## Discussion

### Taphonomy of FMNH PR 3847

#### Geology

Several authors provide detailed geologic descriptions of the Mussentuchit Member [19,88,89]. FMNH locality UT080821-1 is positioned approximately 30 meters from the base of the Cedar Mountain Formation, which in this outcrop includes only the Buckhorn Conglomerate, Ruby Ranch, and Mussentuchit members.

**Fig 10:**
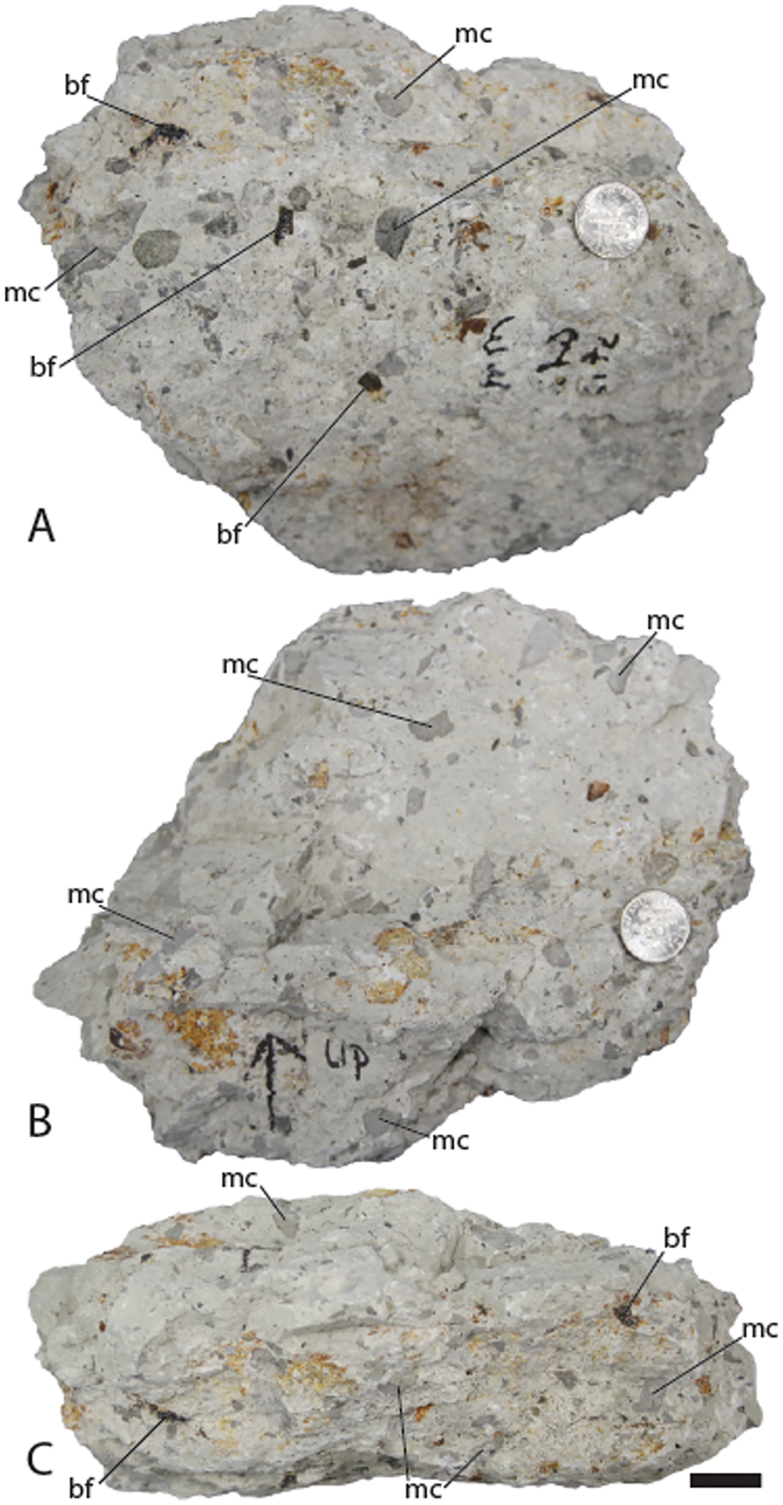
Sedimentary matrix entombing fossils from FMNH Locality UT080821-1. (A) Bottom view of sediment sample showing texture from near base of entombing layer. Note the black bone and higher amount of grey rip-up clasts. (B) Top view of sediment sample showing texture from near top of entombing layer. Note the near absence of bone, fewer rip-up clasts and generally cleaner appearance of the sediment. (C) Side view of sediment sample showing vertical change in entombing layer. Sample is oriented with the upper layer pointed toward to top of the figure. *Abbreviations*: *bf*, bone fragment; *mc*, mud clast. Scale bar equals 18 mm.

The locality resides in an approximately two-meter coarsening upward sequence starting approximately one meter below the base of the bone-bearing horizon with a freshwater limestone abruptly changing to a grey fine-grained mudstone. This layer is truncated by a coarse-grained, white sandstone containing large rip-up clasts of the underlying grey mudstone (Fig 10). The sandstone lens fines upwards to a sharp contact with another whitish-grey silt/mudstone. As is common throughout much of the Mussentuchit Member, the FMNH Locality UT080821-1 sandstone contains a large amount of clay, presumably from altered volcanic ash.

#### Microvertebrate assemblage

**Fig 11:**
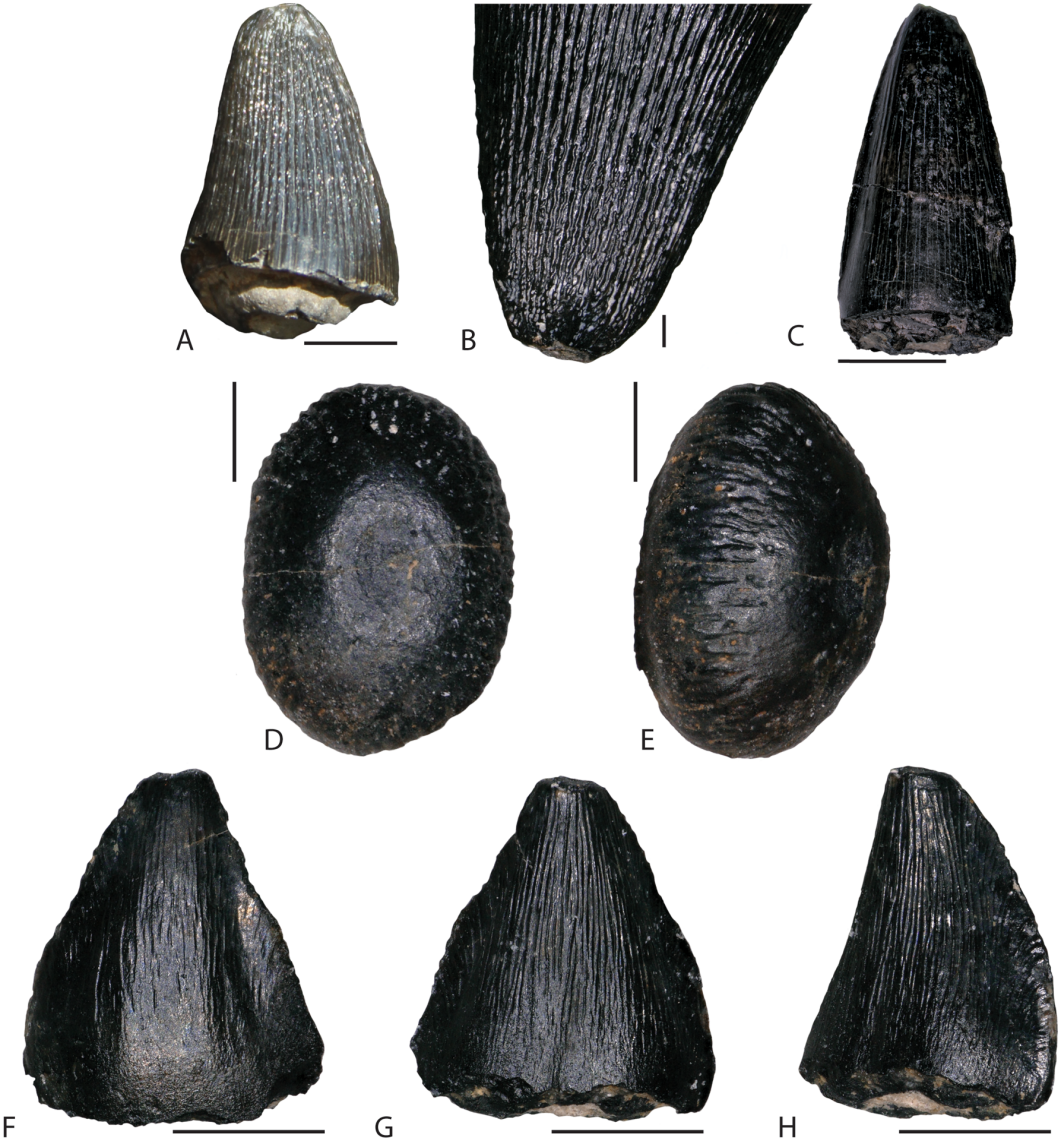
Neosuchian crocodylomorph teeth from FMNH Locality UT080821-1. (A) “pholidosaur”-type FMNH PR 3845. (B) FMNH PR 3845 magnified to show longitudinal ridges on crown. (C) “pholidosaur”-type FMNH PR 3846 showing unornamented crown with lateral carina. “Globidontid”-morphotype FMNH PR 3843 in dorsal (D) and dorsolateral oblique (E) views. “Atoposaurid”-type tooth FMNH PR 3844 in labial (F), lingual (G), and lingual oblique (H) views. All images except B were taken with a Keyence VCX-5000 digital microscope. Image in C was processed through the Keyence VCX-5000 High Dynamic Range filter prior to photographing in order to reduce glare and illuminate specimen detail. Scale bars in A and C equal 5 mm. Scale bars in B and D–H equal 1 mm.

In addition to the partial *Eolambia* skeleton, hundreds of microfossils have been recovered from the site, almost exclusively referable to neosuchian crocodylomorphs. Teeth dominate this microvertebrate assemblage, although some postcranial and possible cranial elements from small neosuchian individuals are present. Generic identification of the crocodylomorph fauna from the Mussentuchit Member is still tenuous. Prior authors [19,28,90] hypothesized at least three groups of crocodilians based on tooth morphology: “atoposaurid”, “globidontid/bernissartid” (two species of *Bernissartia* [19]), and “pholidosaurid”. Recently though, Irmis [91] clarified that the general dental traits used to identify each of these groups could not reliably be used for taxonomic purposes. Therefore, we classify the crocodilians strictly as Neosuchia indet. (Fig 11).

The largest teeth in the sample are what have been considered pholidosaurid, being broad and round at the base with straight longitudinal ridges extending from the base to the tip (Fig 11A and 11B). Another “pholidosaurid” tooth morphology from our sample is smooth-sided apart from two carinae on the lateral portions of the lingual surface (Fig 11C). The “globidontid” morph consists of ovoid teeth with minor pitting and ridges on the edges. Both of the latter two tooth morphotypes have constricted bases unlike the “pholidosaurid” form (Fig 11C and 11D). Finally, the “atoposaurid”-type teeth are labio-lingually compressed, triangular, and highly decorated with irregularly-spaced and non-linear longitudinal ridges (Fig 11F–11H). In many cases, such as FMNH PR 3844, the tips curve lingually.

One important aspect of this quarry is that almost all of the neosuchian material is small. A small neosuchian femur, FMNH PR 3028, is the largest non-tooth element recovered, at 53 mm maximum length. As such, it seems reasonable that these elements might be referable to the same species. If this supposition is true, then some of the “atoposaurid” and “globidontid”-type teeth might actually belong to the same neosuchian taxon. This observation is important because no complete crocodylomorph jaw elements with dentition have been described from the Mussentuchit Member. If true, this follows the observation of Irmis [91] that in other species these two morphotypes co-occur.

**Fig 12:**
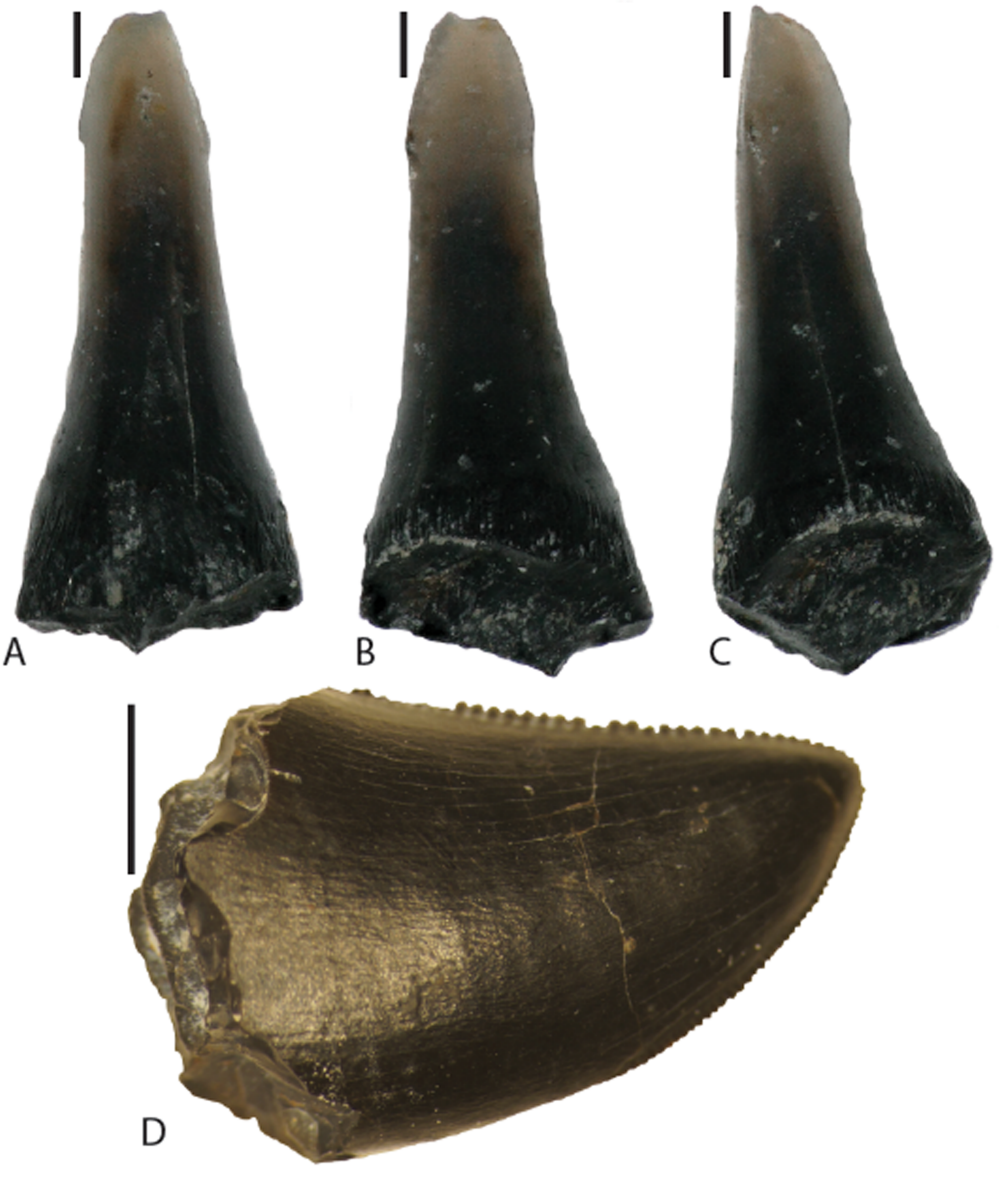
Gar and theropod teeth from FMNH Locality UT080821-1. Gar tooth FMNH PF 15907 in labial (A), lingual (B), and oblique (C) views. Note the general lanceolate shape of the tip and the lingually-oriented concave depression. Specimens A–C were taken with a Keyence VCX-5000 digital microscope. (D) Theropod dinosaur tooth? *Siats meekerorum* FMNH PR 4255 in labial view. Scale bars in A–C equal 0.2 mm. Scale bar in D equals 5 mm.

Non-crocodylomorph microvertebrate fossils recovered from FMNH Locality UT080821-1 include a single gar tooth (FMNH PR 15907; Fig 12A–12C) and theropod teeth provisionally referred to? *Siats meekerorum* [30] based on provenance and size of teeth from other nearby localities with the same serration densities (Fig 12D). The gar tooth possesses a spatulate morphology similar to species of *Atractosteus* [92] and other Late Cretaceous examples from Utah [93], although it differs in being lingually concave. It is unclear if the distal-most point is blunted because of breakage or if this morphology is inherent to the species.

Importantly, FMNH PR 15907 is one of the earliest definitive gar specimens in North America. Garrison et al. [19] figured plausible gar scales from the Cifelli-2 site, a locality near FMNH Locality UT080821-1, but scales are less diagnostic than teeth.

#### Taphonomic interpretation

Based on current geological evidence, we interpret FMNH Locality UT080821-1 as a crevasse splay burial of a partial *Eolambia* skeleton (FMNH PR 3847) on a floodplain. Proximity to the river is unknown, but given the size of the rip-up clasts we assume that the levee breakage was relatively close to the skeleton.

The provenance of the crocodylomorph material remains unclear. The tiny size of the preserved elements might indicate that the floodwaters excavated a buried nest, but this would not explain the disarticulation of elements prior to burial. Nonetheless, the preponderance of crocodylomorph bones compared to the paucity of other species would suggest at least a gathering of small crocodylomorph skeletons prior to the flood event, and not a random sampling of the local microvertebrate fauna.

### Histological description and age estimation of FMNH PR 3847

**Fig 13:**
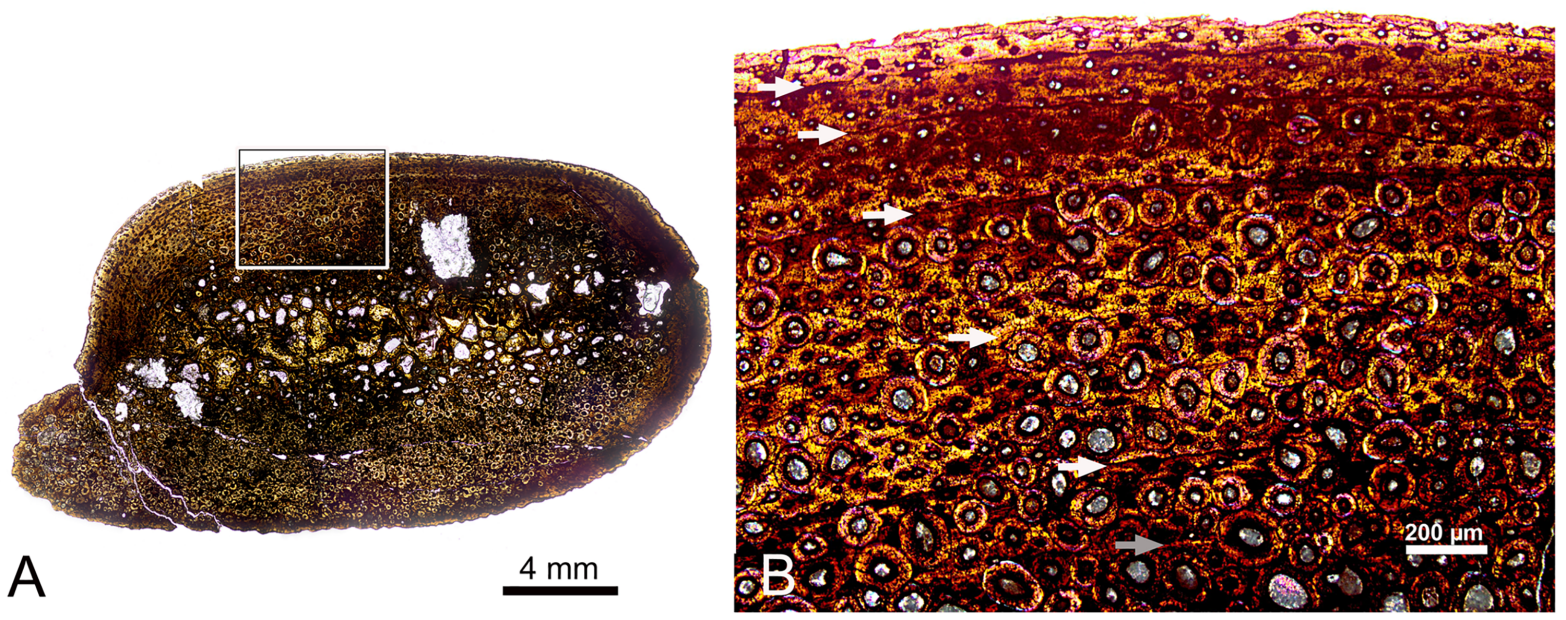
Histology of FMNH PR 3847. (A) Composite micrograph of cross section of caudal dorsal rib of FMNH PR 3847 stitched from 42 individual micrographs. The boxed area is magnified in (B) under polarized light. Five definitive LAGs are marked with white arrows, while a sixth potential growth mark (grey arrow) may be present, but this is less certain as it is truncated by secondary osteons.

The histological cross section of the caudal dorsal rib exhibits extensive secondary remodeling of the bone matrix. The medullary region is marked by numerous, irregular erosional lacunae, and the lateral side of the rib shaft exhibits almost complete replacement of the bone interior by Haversian systems, with some secondary osteons crosscutting the boundaries of prior secondary osteonal growth. The inner surface of the rib shaft is less remodeled preserving zonal bone with primary osteons and growth marks, particularly in the caudomedial region (Fig 13), although scattered secondary osteons are present. Five Lines of Arrested Growth (hereafter LAGs) are clearly visible in the widest preserved band of zonal bones (white arrows in Fig 13B); all are truncated, but the outermost are truncated by remodeling toward the lateral side of the rib. Traces of a probable sixth growth line are observed in the Haversian bone in the medullary region (grey arrow in Fig 13B), but have very little lateral extent so identification of this feature as an annual marker is less certain. Spacing between definitive LAGs decreases noticeably toward the periphery indicating that growth in the rib was slowing, but presence of primary osteons in the outermost zone suggest that an External Fundamental System indicative of growth cessation was not yet being formed when the individual died.

Observed growth markers provide a minimum age of five years for this individual, or six years if the probable growth marker in the medullary region is counted. All of these growth marks are located in the outer 50% of the bone cortex as measured along a transect extending between the long axis of the cross section and perpendicular to the tangent to the external bone surface above the section of the rib preserving the best growth record (Fig 13), so these ages clearly represent an underestimate. Sophisticated mathematical methods have been proposed for retrocalculation of growth marks lost to remodeling and medullary cavity expansion [35,94], but these require that growth marks can be traced along the full circumference of the bone, something that is not possible with the sectioned rib of FMNH PR 3847. Instead, we took a simplified approach to estimating the number of growth markers that have been erased by secondary remodeling by measuring the distance along the transect defined above from the long axis of the bone cross section to the innermost definitive LAG, and dividing by the greatest distance measured between observed LAGs along the same transect defined above (Fig 13). This rough calculation indicates 4.3 missing growth markers. Rounding down to account for the hatching size of the rib, we conservatively estimate that three or four growth markers were lost to secondary remodeling, suggesting an age of eight or nine years at death for this individual.

Histological analysis of the trunk rib shaft indicates that FMNH PR 3847 was still growing at the time of death, an observation that is consistent with other skeletochronological evidence such as the unfused and even fully open neurocentral sutures in the presacral column [95] and lack of fusion between sacral centra and ribs. The examined rib section exhibits decreasing spacing between LAGs toward the periphery, indicating a slow-down in rib growth. Without limb bones for FMNH PR 3847, it is difficult to ascertain whether the slow-down in rib growth mirrors patterns in faster growing limb elements. Horner et al. [33] found a greater possible number of annual lines in the EFS of a rib than in those of hind limb bones in a somatically mature specimen of *Hypacrosaurus stebingeri*, suggesting that ribs may cease growing before limb bones do in hadrosaurs.

FMNH PR 3847 is still a growing subadult with an estimated age of eight to nine years based on five observed and three to four retrocalculated growth markers. This is slightly older than the mean age for attainment of somatic maturity in *Maiasaura* [36], but still within the observed range of individual of growth trajectories for that taxon (Figure 2 in [36]), and also comparable to results published for a mature specimen of *Hypacrosaurus stebingeri* [33], which records five to six LAGs in rib sections, but seven to eight LAGs in the tibia prior to deposition of an EFS in each bone.

### Phylogenetic implications

**Fig 14:**
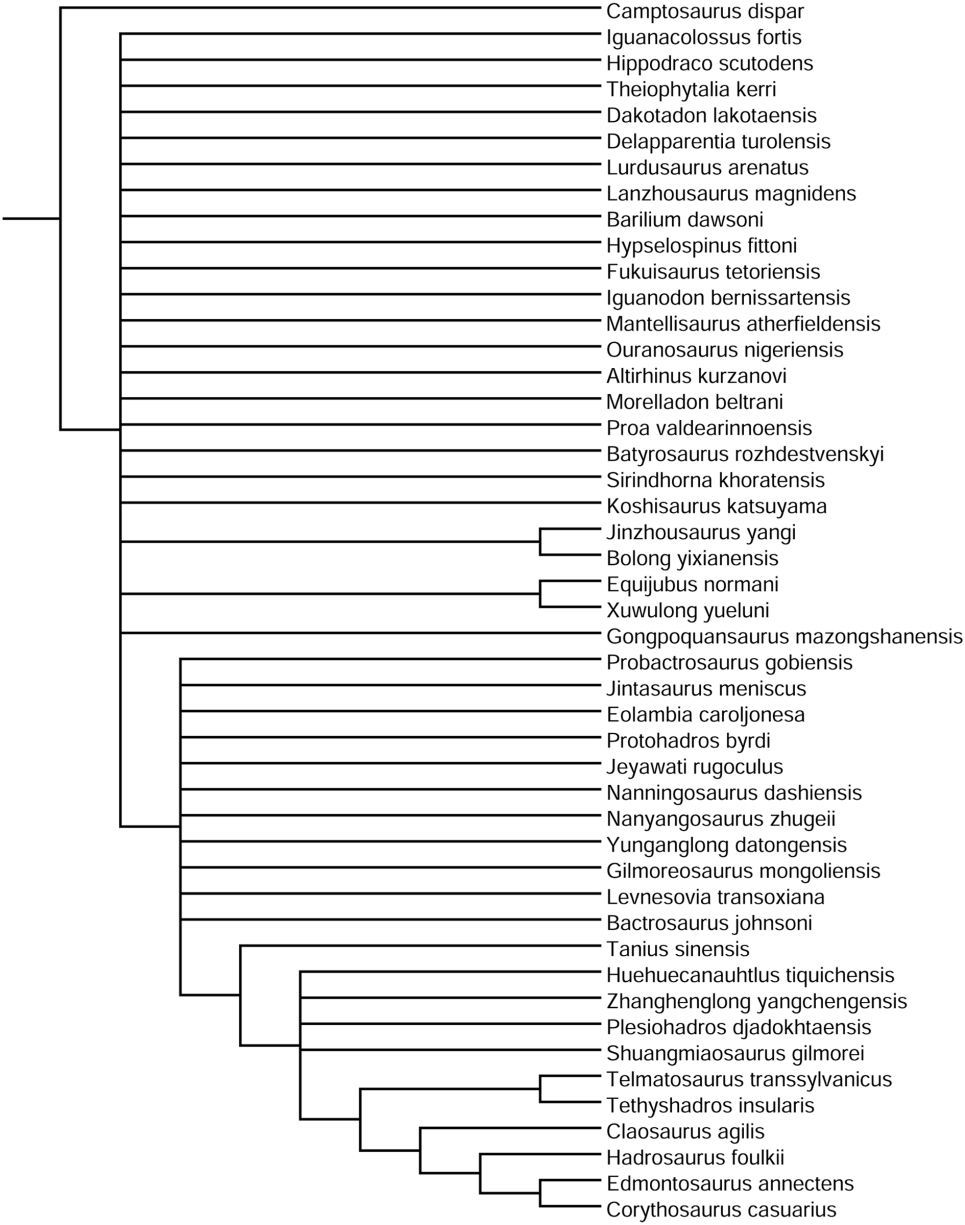
Phylogenetic relationships of *Eolambia caroljonesa*. Strict consensus of 2,060 most parsimonious trees.

**Fig 15:**
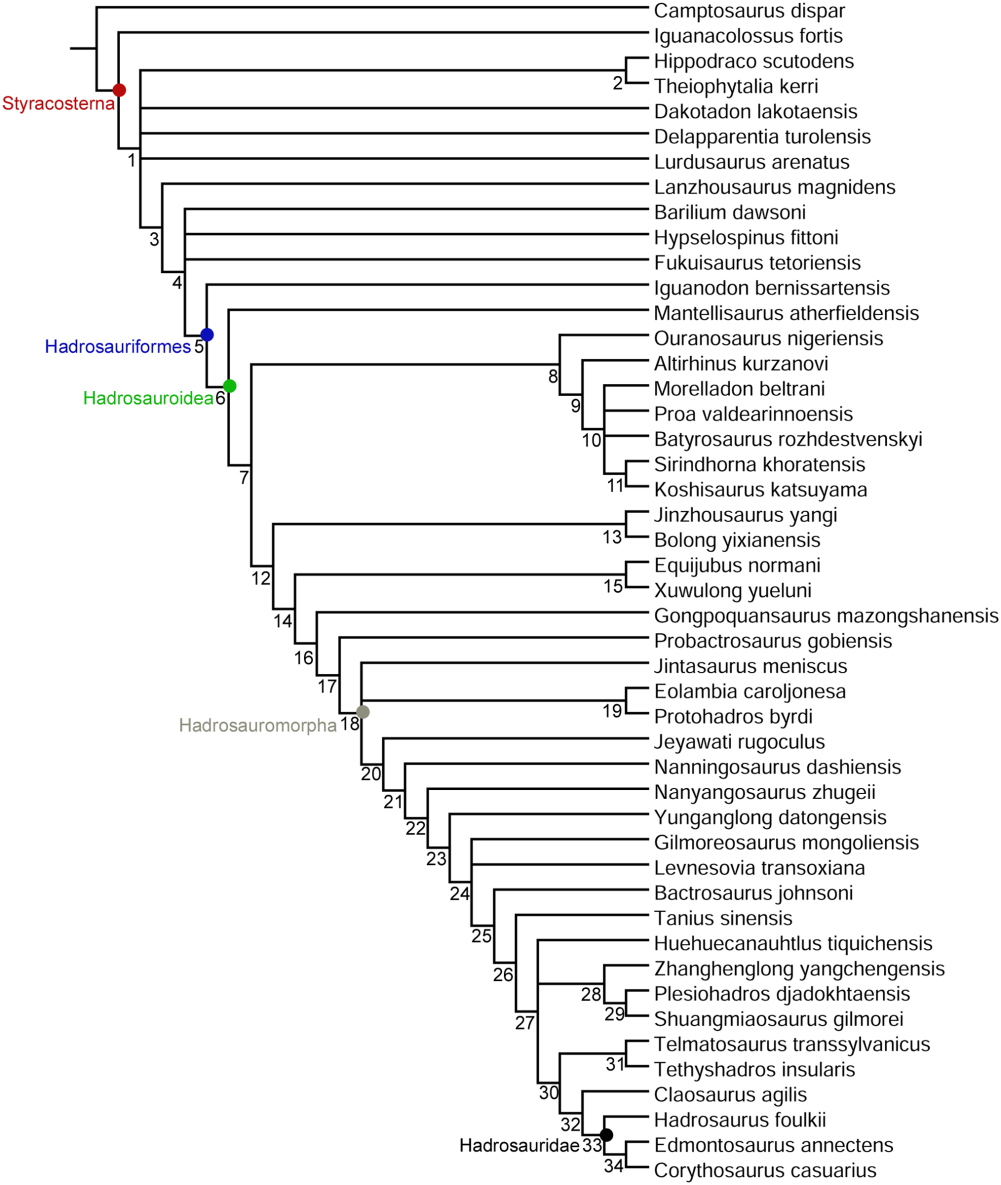
Phylogenetic relationships of *Eolambia caroljonesa*. 50% majority rule consensus of 2,060 most parsimonious trees. Node numbers correspond to those in S3 (S3, List of Synapomorphies).

The strict consensus cladogram is poorly resolved (Fig 14); therefore, we base our discussion on the 50% majority rule consensus cladogram, which has much greater resolution (Fig 15). Although the overall stepwise pattern of non-hadrosaurid styracosternan evolution is consistent with previous analyses [4,8,13,15,16,21,58,60,63,65,96,97], there are a number of intriguing small clades that emerge in this revised analysis.

Most of these clades are geographically restricted and suggest that brief periods of endemism might have been a recurring phenomenon throughout styracosternan evolution. As in prior versions of this analysis, *Hippodraco* and *Theiophytalia* form a clade of basal styracosternans restricted to the Barremian–Albian? of western North America [4,98]. The analysis recovered two small basal hadrosauroid clades restricted to the Aptian (*Jinzhousaurus* and *Bolong* [5,63,82]) and Albian (*Equijubus* and *Xuwulong* [68,69,96]) of east-central Asia.

This pattern of endemism continued into the Late Cretaceous. *Eolambia* itself and *Protohadros* comprise a clade of basal hadrosauromorphs from the Cenomanian of west-central North America [19,20,21]. *Shuangmiaosaurus*, *Zhanghenglong*, and *Plesiohadros* form a clade of derived non-hadrosaurid hadrosauromorphs from the? Cenomanian–Campanian of east-central Asia [11,16,97]. Finally, *Telmatosaurus* and *Tethyshadros* comprise a clade of dwarf hadrosauromorphs restricted to the Campanian–Maastrichtian of the European Archipelago [71,99].

In contrast to these small, geographically and temporally restricted clades, a widespread and long-lived novel clade of basal hadrosauroids also was recovered by this analysis. This clade consists of taxa from North Africa (*Ouranosaurus* [59]), Europe (*Morelladon* [100] and *Proa* [41]), and Asia (*Altirhinus* [67], *Batyrosaurus* [14], *Koshisaurus* [45], and *Sirindhorna* [101]). Most members of this clade are of Early Cretaceous age (Barremian–Albian); however, if the Santonian age of *Batyrosaurus* proposed by Godefroit et al. [14] is accurate, then this clade persisted into the Late Cretaceous. There are several theropod clades that exhibit similar geographic and temporal distributions, with members found in the Early and Late Cretaceous of North Africa, Europe, and Asia, including Spinosauridae [102], Carcharodontosauridae [103,104,105], and Neovenatoridae [30,106,107].

It should be noted that Verdú et al. [108] recently and independently conducted a phylogenetic analysis using a prior version of the same basic data matrix employed herein. There are some noteworthy similarities between our results and those of Verdú et al. [108], such as a Late Cretaceous “Asian clade” including *Shuangmiaosaurus*, *Zhanghenglong*, and *Plesiohadros* (Fig 15). Verdú et al. [108] also recovered a Late Cretaceous “North American clade” that included *Eolambia* and *Protohadros*; however, their analysis placed *Jeyawati* in this clade, while our results position *Jeyawati* as a more derived hadrosauromorph (Fig 15). Finally, Verdú et al. [108] recovered a “Eurasian clade” similar to the clade of North African, European, and Asian taxa in our results. However, the clade found by Verdú et al. [108] excluded *Ouranosaurus* and *Sirindhorna*, but included *Xuwulong* and *Gongpoquansaurus*, which we find to be more derived hadrosauroids (Fig 15). The phylogenetic and biogeographic history of Styracosterna is clearly very complex and will require much additional analysis to refine.

## Supporting information

S1 Spreadsheet Data matrix. Character-taxon matrix used in the phylogenetic analysis.
(XLS)

S2 Spreadsheet List of specimens examined. Iguanodontian specimens examined firsthand.
(XLS)

S1 Document Character list, list of supplemental references, and list of synapomorphies. List of morphological characters and supplemental references used in the phylogenetic analysis, and list of synapomorphies.
(DOC)

S1 File Nexus file formatted for TNT. Nexus file used in the phylogenetic analysis.
(TXT)

S1 Table Table of measurements. Measurements of select anatomical features of FMNH PR 3847.
(DOC)

## Abbreviations

AMNH: American Museum of Natural History, New York, NY, USA
ANSP: Academy of Natural Sciences, Philadelphia, PA, USA
CEUM: College of Eastern Utah Prehistoric Museum, Price, UT, USA
CM: Carnegie Museum of Natural History, Pittsburgh, PA, USA
DMNS: Denver Museum of Nature and Science, Denver, CO, USA
FMNH: Field Museum of Natural History, Chicago, IL, USA
IRSNB: Institut royal des Sciences naturelles de Belgique, Brussels, Belgium
IVPP: Institute of Vertebrate Paleontology and Paleoanthropology, Beijing, China
MIWG: Museum of Isle of Wight Geology (Dinosaur Isle Museum), Sandown, UK
MNHN: Muséum national d’Histoire naturelle, Paris, France
MSM: Arizona Museum of Natural History (formerly Mesa Southwest Museum), Mesa, AZ, USA
NHMUK: The Natural History Museum, London, UK
OMNH: Sam Noble Oklahoma Museum of Natural History, Norman, OK, USA
PIN: Palaeontological Institute, Moscow, Russia
SDSM: South Dakota School of Mines and Technology, Rapid City, SD, USA
SMU: Southern Methodist University Shuler Museum of Paleontology, Dallas, TX, USA
UMNH: Natural History Museum of Utah, Salt Lake City, UT, USA
USNM: National Museum of Natural History, Washington, DC, USA
YPM: Yale Peabody Museum of Natural History, New Haven, CT, USA
