# Supplementary material for: Anatomy, taphonomy, and phylogenetic implications of a new specimen of Eolambia caroljonesa (Dinosauria: Ornithopoda) from the Cedar Mountain Formation, Utah, USA
Source: PLoS One. 2017 May 10;12(5):e0176896. doi: 10.1371/journal.pone.0176896 (PMC5425030; doi:10.1371/journal.pone.0176896)
Supplement: S1 Document — List of morphological characters and supplemental references used in the phylogenetic analysis, and list of synapomorphies. (DOC) [file pone.0176896.s003.doc]

**S1 Document. Character List, Additional References and Synapomorphies**

**Character List**

Description of characters used in the phylogenetic analysis of Iguanodontia. Characters are numbered 0–134 in the format of TNT. Abbreviations refer to specific skeletal elements or regions (e.g., PD1 is predentary character 1): PD, predentary; DT, dentary; SU, surangular; ANG, angular; PM, premaxilla; MX, maxilla, LAC, lacrimal, PRF, prefrontal; PO, postorbital; JG, jugal; QU, quadrate; SQ, squamosal; BC, braincase; T, teeth; AX, axial column; PC, pectoral girdle; FL, forelimb; PV, pelvic girdle; HL, hind limb.

**Mandibular**

0. Predentary, overall shape of oral portion in dorsal view: arcuate, rounded rostrolateral corners (0); subtriangular, comes to a point without a distinct rostral portion (1); subrectangular, squared corners and straight, well demarcated rostral portion (2) (modified from Weishampel et al. 2003, character 18; and Prieto-Márquez et al. 2006b, character 5). PD1

1. Predentary, directions of lateral margins of lateral processes relative to each other in dorsal and ventral views: divergent (0); parallel (1). PD2

2. Predentary, dorsomedial process: present (0); absent (1). PD3

3. Predentary, denticle morphology: large, conical median denticle with one or two prominent conical denticles of subequal size adjacent to the median denticle on both sides and smaller, tab-like denticles on lateral processes (0); rostrocaudally compressed prong-like denticles that increase in size towards the midline of the predentary (1); rostrocaudally compressed prong-like denticles of equal size (2) (modified from Prieto-Márquez 2010, characters 25 and 27) PD4

4. Dentary, orientation of symphysis relative to lateral margin of dentary: rostrolateral to caudomedial (medial edge of symphysis and lateral margin of dentary diverge caudally in dorsal view) (0); parallel (1) (Prieto-Márquez et al. 2006b, character 10). DT1

5. Dentary, diastema: absent (0); present (1). DT2

6. Dentary, shape of tooth row in dorsal view: bowed medially along caudal half (0); straight (1) (modified from Prieto-Márquez et al. 2006b, character 8). DT3

7. Dentary, shape of tooth row in lateral view: straight (0); convex (1). DT4

8. Dentary, orientation of tooth row relative to lateral surface of dentary: convergent rostrally and caudally (0); convergent rostrally and divergent caudally (1). DT5

9. Dentary, morphology of tooth alveoli: alveoli shaped by dentary teeth (0); alveoli with parallel vertical walls (1) (Norman 2002, character 33). DT6

10. Dentary, caudal-most extent of tooth row: medial to coronoid process but still rostral to longitudinal axis of the process (0); caudal to longitudinal axis of the coronoid process but still rostral to the caudal margin of the process (1); caudal to the base of the coronoid process (2) (modified from You et al. 2003b, character 29). DT7

11. Dentary, shape in lateral or medial view: dorsal and ventral margins are parallel (0); deepens rostrally (1) (modified from Norman 2004, character 22). DT8

12. Dentary, morphology of ventral margin of rostral ramus leading to the predentary articulation and symphysis: straight (0); ventral margin inflected ventrally, such that it curves gently towards the predentary articulation and symphysis (1); ventral margin curves dorsally towards symphysis (2). DT9

13. Dentary, bulge along ventral margin directly ventral to the base of the coronoid process: absent (0); present (1) (modified from Prieto-Márquez 2010, character 41). DT10

14. Dentary, bulge on the lateral surface ventral to the coronoid process that gives rise to the process: absent (0); present (1) (modified from Prieto-Márquez 2010, character 46). DT11

15. Dentary, platform between the tooth row and the coronoid process: absent, tooth row curves into base of coronoid process (0); present (1) (modified from Norman 2002, character 26). DT12

16. Dentary, orientation of coronoid process: caudally inclined (0); vertical (1); rostrally inclined (2) (modified from Prieto-Márquez et al. 2006b, character 7). DT13

17. Dentary, expansion of dorsal end of coronoid process: absent (0); present (1). DT14

18. Dentary, expansion of dorsal end of coronoid process, location: along rostral edge only (0); along rostral and caudal edges (1) (modified from McDonald et al. 2010b, character 33). DT15

19. Dentary, position of greatest rostrocaudal width of expanded coronoid process: ventral to apex (0); at apex (1). DT16

20. Surangular, surangular foramen: present (0); absent (1) (modified from Weishampel et al. 1993, character 27). SU1

21. Surangular, external mandibular fenestra: small foramen (“accessory foramen”) on surangular near suture with dentary (0); absent (1) (modified from Kobayashi and Azuma 2003, character 15). SU2

22. Surangular, shape of contact with angular in lateral view: inclined rostrodorsal to caudoventral (0); horizontal (1). SU3

23. Surangular, shape of rostrodorsal process in lateral view: rostrocaudally broad and exposed in lateral view (0); rostrocaudally narrow and strap-like, mostly concealed in lateral view by the caudal margin of the coronoid process (1) (modified from Prieto-Márquez 2010, character 51). SU4

24. Angular, exposure in lateral view: present, groove on ventral margin of surangular for articulation with angular (0); absent, articulation with surangular occurs on the medial surface of that bone (1) (modified from Norman 2002, character 28). ANG1

**Cranial**

25. Premaxilla, morphology of caudolateral corner of oral margin in lateral view: free and gently curved (0); free and angular (1). PM1

26. Premaxilla, everted rim on lateral edge of oral margin: absent (0); present (1) (modified from Weishampel et al. 1993, character 3). PM2

27. Premaxilla, denticle morphology: one large conical denticle adjacent to interpremaxillary suture on each premaxilla (0); two large, rostrocaudally elongate denticles on each premaxilla (1); three or more conical denticles of similar size on each premaxilla (2). PM3

28. Premaxilla, morphology of caudal ramus of ventrolateral process: tapers (0); dorsoventrally expanded (1) (modified from Prieto-Márquez 2010, character 71). PM4

29. Premaxilla, transverse ridge of thickened bone caudal to oral margin, separated from the oral margin by a deep sulcus bearing vascular foramina: absent (0); present (1) (modified from Prieto-Márquez 2010, character 63). PM5

30. Premaxilla, shape of external naris: subcircular, with a rounded caudal margin and deeply embayed ventral margin (0); elliptical, with a tapering caudal margin and gently curved ventral margin (1). PM6

31. Premaxilla, shape of rostroventral part of circumnarial fossa: floor of fossa oriented dorsomedially and not well exposed in lateral view (0); floor of fossa oriented dorsoventrally and extensively exposed in lateral view (1). PM7

32. Maxilla, rostrodorsal process: absent (0); present (1) (modified from Prieto-Márquez et al. 2006b, character 17). MX1

33. Maxilla, direction of rostroventral process: rostrally directed (0); rostroventrally curved (1). MX2

34. Maxilla, ventral margin of tooth row in lateral view: straight (0); concave (1). MX3

35. Maxilla, shape in dorsal view: bowed medially (0); straight for most of length (1); bowed laterally (2). MX4

36. Maxilla, shape of tooth row in ventral view: medially bowed, with rostral and caudal ends curving laterally (0); bowed laterally (1); straight (2). MX5

37. Maxilla, jugal process morphology: sinuous shelf, scarf contact with jugal (0); caudolaterally projecting jugal process, “finger-in-recess” contact with jugal (1); mediolaterally broad, flat surface against which jugal abuts (2) (modified from Norman 2002, character 15). MX6

38. Maxilla, antorbital fossa, extent in lateral view: occupies most of lateral surface of ascending process (0); rostrocaudally elongate, elliptical depression restricted to caudal half of ascending process (1); small semicircular depression restricted to caudal margin of ascending process (2); antorbital fossa not visible in lateral view (3). MX7

39. Maxilla, bifurcation of maxillary ascending process into lateral and medial lacrimal processes: absent (0); present (1). MX8

40. Maxilla, morphology of rostrodorsal process: low eminence on rostrodorsal margin of maxilla (0); prominent, rostrally-projecting prong separated from the rostroventral process by a deep embayment (1). MX9

41. Lacrimal, concave ventral margin to form part of antorbital fenestra: present (0); absent (1). LAC1

42. Lacrimal, morphology of rostral ramus: tapers to a point (0); dorsoventrally expanded (1). LAC2

43. Lacrimal, morphology of ventral ramus: tapers to a point (0); rounded (1). LAC3

44. Lacrimal, contact with nasal: present (0); absent (1) (Norman 2002, character 12). LAC4

45. Prefrontal, morphology of nasal process: tapering, finger-like projection (0); dorsoventrally broad, mediolaterally compressed plate (1). PRF1

46. Postorbital, shape of caudal end of squamosal process that overlaps the lateral surface of the squamosal: rounded (0); bifurcated (1) (modified from Prieto-Márquez 2010, character 132). PO1

47. Postorbital, lateral surface: smooth, rugosity restricted to orbital rim (0); rugosity on dorsolateral surface (1) (modified from Godefroit et al. 2009). PO2

48. Postorbital, length of squamosal process: short, postorbital-squamosal contact does not reach caudal margin of supratemporal fenestra (0); long, contact reaches the caudal margin of the supratemporal fenestra (1) (modified Prieto-Márquez et al. 2006b, character 37). PO3

49. Jugal, articulation with ectopterygoid: present (0); absent (1) (Head 1998, character 6). JG1

50. Jugal, morphology of portion of maxillary process that overlaps maxilla: tapers at rostral ends of maxillary and lacrimal contacts, with slightly convex ventral margin and slightly concave dorsal margin (0); tapers with sinuous dorsal and ventral margins (1); dorsoventrally expanded (2); dorsoventrally expanded to form part of rostral margin of orbit (3) (modified from Norman 2002, character 14). JG2

51. Jugal, large neurovascular foramen at base of postorbital process on medial surface: absent (0); present (1). JG3

52. Jugal, shape of free ventral margin caudal to maxillary contact: sinuous, jugal dorsoventrally expanded ventral to infratemporal fenestra (0); sinuous with striated, caudally-directed flange that projects caudal to jugal-quadratojugal contact (1); angular, with prominent ventrally-directed flange ventral to infratemporal fenestra (2); dorsoventrally narrow and strap-like, with convex ventral margin and concave dorsal margin that are parallel (3) (modified from Norman 2002, character 16). JG4

53. Jugal, articulation with jugal process of postorbital: postorbital process of jugal notched at dorsal end (0); postorbital process of jugal bears an elongated facet on its cranial margin (1) (modified from Head 1998, character 7). JG5

54. Jugal, relative widths of the orbital and infratemporal margins in lateral view: orbital margin wider (0); infratemporal margin wider (1) (modified from Prieto-Márquez 2010, character 115). JG6

55. Quadrate, shape of notch in lateral wing: semicircular (0); broad and crescentic (1) (modified from Prieto-Márquez et al. 2006b, character 40). QU1

56. Quadrate, paraquadrate foramen: present, gap between portion of caudal margin of quadratojugal and rostral margin of quadrate (0); absent, caudal margin of quadratojugal contacts entire rostral margin of quadrate along the contact surface (1). QU2

57. Quadrate, overall shape in lateral or medial view: straight for much of dorsoventral length, curved caudally near dorsal end (0); curved gently caudally along entire length (1); straight (2). QU3

58. Quadrate, shape of dorsal condyle: subtriangular, broad rostral margin and tapers to a point caudally (0); D-shaped, broadest along lateral profile (1) QU4

59. Quadrate, shape of ventral condyle: rostrocaudally narrow and mediolaterally broad, with larger lateral condyle, and medial condyle slopes ventrolaterally towards lateral condyle (0); distinct step between medial condyle and larger, more ventrally situated lateral condyle (1). QU5

60. Squamosal, orientation of caudomedial process: curved rostromedially (0); straight and medially directed (1) (modified from Prieto-Márquez et al. 2006b, character 45). SQ1

61. Squamosal, relationship of right and left squamosals on skull roof: widely separated by parietal (0); separated by only a narrow band of the parietal (1); in broad contact with each other (2) (Horner et al. 2004, character 63). SQ2

62. Frontal, participation in dorsal orbital rim: present (0); absent (1) (Norman 2002, character 19). BC1

63. Supraoccipital, contribution to foramen magnum: present (0); absent, excluded by exoccipitals (1) (You et al. 2003b, character 23). BC2

64. Supraoccipital, morphology of supraoccipital-exoccipital contact: straight suture that meets squamosal (0); ventrolateral corner of supraoccipital is inset into exoccipital so that supraoccipital is locked between exoccipitals (1) (Horner et al. 2004, character 66). BC3

65. Supraoccipital, inclination of caudal surface: caudal surface rostrally inclined (0); caudal surface vertical (1) (modified from Horner et al. 2004, character 65). BC4

66. Exoccipital-Opisthotic, paroccipital process orientation of pendant distal portion: straight and ventrally directed (0); curved rostrally (1) (Horner et al. 2004, character 62). BC5

67. Basioccipital, orientation of occipital condyle: caudoventrally directed (0); caudally directed (1) (modified from Prieto-Márquez 2010, character 152). BC6

68. Basioccipital, rostrocaudally directed groove extending along ventral surface: present (0); absent (1). BC7

69. Basioccipital, morphology of surface between basal tubera: broad, shallow trough with ridge extending rostrocaudally down the midline (0); broad, shallow trough with smooth floor (1). BC8

70. Basisphenoid, surface between basipterygoid processes: transverse, sharply defined ridge between basipterygoid processes (0); ventrally directed prong between basipterygoid processes (1) (modified from Gates and Sampson 2007, characters 78 and 79). BC9

71. Foramen magnum, composition of ventral margin: caudomedial surfaces of left and right exoccipitals and dorsal margin of basioccipital (0); left and right exoccipitals only (1) (modified from Weishampel et al. 1993, character 24). BC10

72. Parietal, shape of sagittal crest in lateral view: approximately level with the skull roof (0); deepens caudally (1) (modified from Prieto-Márquez 2010, character 148). BC11

73. Parietal, rostral extent of the sagittal crest: extends along entire length of parietal and is sharply-defined at rostral end (0); extends along entire length of parietal but diminishes near rostral end (1); extends along only the caudal half of the parietal (2) (modified from Prieto-Márquez 2010, character 150). BC12

74. Basioccipital, distinct neck between basal tubera and occipital condyle: present (0); absent (1) (Godefroit et al. 2009). BC13

75. Infratemporal fenestra, location of dorsal margin relative to dorsal margin of orbit: more ventrally located (0); approximately at same level (1); more dorsally located (2) (modified from Prieto-Márquez 2010, character 192). BC14

76. Supratemporal fenestra, shape in dorsal view: oval, long axis directed rostrally (0); oval, long axis directed rostrolaterally (1); oval, long axis oriented mediolaterally (2) (modified from Prieto-Márquez 2010, character 193). BC15

**Dentition**

77. Dentary teeth, morphology of marginal denticles: tongue-shaped with smooth edges (0); tongue-shaped with mammillated edges (1); reduced to small mammillated papillae (2) (modified from Norman 2002, character 31). T1

78. Dentary teeth, number of replacement teeth per tooth position: one (0); two (1); three (2) (modified from Weishampel et al. 1993, character 32). T2

79. Dentary teeth, number of teeth per tooth position forming part of occlusal plane: one (0); two (1); three (2) (modified from Norman 2002, character 39). T3

80. Dentary teeth, shape of crown in lingual view: mesiodistally broad, oblong, shield-like surface (0); mesiodistally narrow and diamond-shaped (1) (modified from Norman 2002, character 29). T4

81. Dentary teeth, position of primary ridge: distally offset (0); no offset, primary ridge divides the lingual side of the crown into equal halves (1) (modified from You et al. 2003b, character 39). T5

82. Dentary teeth, number and morphology of ridges on lingual surface of crown: parallel and similarly prominent primary and secondary ridges with multiple faint accessory ridges arising from marginal denticles (0); prominent primary ridge and multiple separate faint accessory ridges to either side of it (1); primary ridge and a single less prominent accessory ridge on either side (2); primary ridge and a single mesial accessory ridge (3); primary ridge only (4). T6

83. Maxillary teeth, number of teeth per tooth position forming part of occlusal plane: one (0); two (1). T7

84. Maxillary teeth, primary ridge position and morphology: distally offset (0); no offset, primary ridge divides the labial side of the crown into equal halves (1) (modified from You et al. 2003b, character 36). T8

85. Maxillary teeth, number and morphology of ridges on labial surface of crown: primary ridge with multiple parallel accessory ridges on either side (0); primary ridge and only mesial accessory ridges (1); primary ridge only (2). T9

**Postcranial (Axial)**

86. Axis, morphology of axial neural spine in lateral view: caudodorsally-sloping process (0); dorsally expanded, convex process (1). AX1

87. Cervical vertebrae, opsisthocoely of centra: slightly opisthocoelous, flat or slightly convex cranial face (0); deeply opisthocoelous, hemispherical cranial face protrudes beyond ventral and dorsal surfaces of centrum and has a smooth, rounded surface (1). AX2

88. Dorsal vertebrae, extremely tall neural spines, more than three times centrum height: absent (0); present (1) (modified from Norman 2002, character 41). AX3

**Postcranial (Appendicular)**

89. Sternal, caudolateral process: absent (0); present (1) (modified from Kobayashi and Azuma 2003, character 23). PC1

90. Sternal, caudomedial process: absent (0); present (1). PC2

91. Sternal, shape of main body in dorsal or ventral view, excluding caudolateral process if present: convex medially and concave laterally (0); convex medially and straight laterally (1). PC3

92. Scapula, dorsal margin of scapular shaft at approximately mid-shaft between acromion process and caudodorsal margin: straight (0); convex (1). PC4

93. Scapula, expansion of caudal end: gently convex expansion along caudodorsal margin, caudoventral margin tapers into hook-like flange (0); caudal end paddle-shaped, dorsal and ventral margins of scapula diverge towards caudal end (1); caudal margin of scapula straight, dorsal and ventral margins are parallel approaching caudal margin of scapula and meet caudal margin at nearly right angles (2). PC5

94. Scapula, acromion process orientation: dorsally directed (0); laterally directed (1) (Norman 2002, character 44). PC6

95. Scapula, morphology of deltoid ridge: dorsoventrally narrow with a poorly demarcated ventral margin, restricted to proximal part of scapula (0); dorsoventrally narrow and sharply defined, with a well-demarcated ventral margin (1); dorsoventrally deep and craniocaudally elongated, with a well-demarcated ventral margin (2) (modified from Prieto-Márquez 2010, character 218). PC7

96. Coracoid, shape of cranial margin: convex (0); straight (1) (modified from Horner et al. 2004, character 78). PC8

97. Humerus, shape of deltopectoral crest: distal margin rounded and merges gradually with the lateral margin of the humeral shaft (0); distal margin angular and merges abruptly with the lateral margin of the humeral shaft (1) (modified from Weishampel et al. 1993, character 37). FL1

98. Manus, digit I: present (0); absent (1) (Norman 2002, character 51). FL2

99. Manus, arrangement of metacarpals II-IV: spreading (0); closely appressed (1) (You et al. 2003b, character 49). FL3

100. Manus, unguals of digits II and III, shape: claw-like (0); flattened and hoof-like (1) (Norman 2002, character 53). FL4

101. Ilium, preacetabular process, cranial end: rounded (0); dorsoventrally-expanded boot offset from shaft (1). PV1

102. Ilium, dorsal margin above pubic and ischial peduncles and acetabulum: straight (0); convex (1); sinuous, convex above pubic peduncle and concave above ischial peduncle (2) (modified from Weishampel et al. 2003, character 55). PV2

103. Ilium, morphology of dorsal margin of postacetabular process dorsal to ischial peduncle: mediolaterally thickened dorsal margin compared to dorsal margin above pubic peduncle (0); thickened and laterally-bulging everted rim along dorsal margin (1); dorsal margin thickened and expanded ventrolaterally to form rounded knob (2); dorsally-projecting flange extending from above acetabulum to above ischial peduncle (3); laterally-projecting, non-pendant supraacetabular process continuous with dorsal margin of ilium (4); pendant supraacetabular process continuous with dorsal margin of ilium (5) (modified from Norman 2002, character 56). PV3

104. Ilium, postacetabular process, shape in lateral view: tapers to a point with break in slope along dorsal margin, forming a distinct platform for the origin of *M. iliocaudalis* (0); tapers with no break in slope along dorsal margin (1); tapers, process curves dorsally along its entire length, such that both the dorsal and ventral margins curve dorsally (2); subrectangular with no break in slope (3) (modified from Norman 2002, character 57). PV4

105. Ilium, curvature of preacetabular process near its base: curves ventrally, with a convex dorsal margin and concave ventral margin (0); straight, with little or no change in slope between its dorsal margin and the dorsal margin of the body of the ilium (1). PV5

106. Ilium, preacetabular process projects ventral to the pubic peduncle: absent (0); present (1). PV6

107. Ilium, morphology of the pubic peduncle: cranioventrally-directed, craniocaudally narrow process (0); craniocaudally broad, subtriangular prominence (1) (modified from Prieto-Márquez 2010, character 241). PV7

108. Ilium, shape of acetabulum: deep, semicircular, dorsal margin strongly arched (0); shallow and crescentic (1). PV8

109. Ilium, morphology of ischial peduncle: ventrolaterally-directed oval prominence (0); oval prominence with smaller prominence on the caudodorsal margin (1); composed of two prominences of similar size (2) (modified from Prieto-Márquez 2010, character 242). PV9

110. Ilium, position of ventral-most point on supraacetabular process relative to the caudal-most point on the ischial peduncle: dorsal (0); cranial (1) (modified from Prieto-Márquez 2010, character 235). PV10

111. Ilium, shape of the supraacetabular process in lateral view: asymmetrical (0); symmetrical (1) (modified from Prieto-Márquez 2010, character 238). PV11

112. Pubis, distal expansion of cranial pubic process: absent, dorsal and ventral margins parallel (0); present, dorsal and ventral margins diverge distally (1) (modified from Norman 2002, character 58). PV12

113. Pubis, caudal pubic process: approximately equal in length to ischium (0); shorter than ischium (1) (Norman 2002, character 59). PV13

114. Pubis, caudal pubic process, morphology of distal end: rounded (0); tapers to a point (1). PV14

115. Pubis, shape of dorsoventral expansion of cranial pubic process: asymmetrical, dorsal portion more expanded than ventral, expansion is directed craniodorsally (0); asymmetrical, ventral portion more expanded than dorsal, expansion is directed cranioventrally (1); symmetrical, dorsal and ventral portions approximately equal in size (2) (modified from Prieto-Márquez 2010, character 252). PV15

116. Pubis, craniocaudal length of the proximal constriction of the cranial pubic process relative to the length of the dorsoventral expansion: constriction is longer (0); approximately the same length (1); constriction is shorter (2) (modified from Prieto-Márquez 2010, character 255). PV16

117. Pubis, shape of proximal constriction of expanded cranial pubic process: dorsal and ventral margins parallel (0); maximum ventral concavity located more proximal than maximum dorsal concavity (1); maximum ventral concavity located approximately ventral to the maximum dorsal concavity (2) (modified from Prieto-Márquez 2010, character 256). PV17

118. Pubis, obturator foramen: present, enclosed by ischial peduncle and caudodorsal process on caudal pubic process (0); absent, no caudodorsal process on caudal pubic process (1) (modified from Prieto-Márquez 2010, character 258). PV18

119. Ischium, morphology of shaft: curved cranially (0); curved caudally (1); straight (2) (modified from Norman 2002, character 60; Weishampel et al. 2003, character 60). PV19

120. Ischium, morphology of distal end: rounded expansion (0); cranially expanded boot (1); bluntly truncated (2) (modified from Prieto-Márquez et al. 2006b, character 126). PV20

121. Ischium, alignment of long axis of shaft: aligned with bisector between the pubic and iliac peduncles (0); aligned with pubic peduncle (1) (Gasca et al. 2014). PV21

122. Ischium, relative proximodistal lengths of the pubic and iliac peduncles in lateral view: pubic peduncle longer (0); iliac peduncle longer (1); lengths subequal (2) (Gasca et al. 2014). PV22

123. Ischium, relative craniocaudal depths of pubic and iliac peduncles in lateral view: iliac peduncle deeper (0); pubic peduncle deeper (1); depths subequal (2) (Gasca et al. 2014). PV23

124. Ischium, acetabular margin in lateral view: broad and crescentic (0); narrow, U-shaped (1) (modified from Gasca et al. 2014). PV24

125. Ischium, curvature between iliac peduncle and shaft of ischium: gentle curve (0); nearly right-angle (1) (modified from Gasca et al. 2014). PV25

126. Ischium, lip projecting from caudal margin of the articular surface of the iliac peduncle: absent (0); present (1). PV26

127. Femur, curvature of shaft in lateral or medial view: distal half of shaft curved caudally (0); distal half of shaft straight (1) (Norman 2002, character 62). HL1

128. Femur, groove on caudal aspect of femoral head: present (0); absent (1) (Winkler et al. 1997, character 25). HL2

129. Femur, morphology of fourth trochanter: pendant (0); broad and triangular (1); curved, mediolaterally compressed eminence (2) (Norman 2002, character 63). HL3

130. Femur, intercondylar extensor groove: deep, narrow, U-shaped, partially enclosed by slight expansion of medial condyle (0); deep, U-shaped, partially enclosed by expansion of medial and lateral condyles (1); canal fully enclosed by lateral and medial condyles (2) (modified from Norman 2002, character 64; Barrett et al. 2011, character 127). HL4

131. Femur, deep cleft separating the greater and cranial trochanters: present (0); absent, lesser trochanter is closely appressed to the proximal end of the femur (1) (modified from Barrett et al. 2011, character 134). HL5

132. Tibia, shape and extent of cnemial crest on cranial margin: cranially-expanded subtriangular flange restricted to proximal end of tibia (0); further extended along the cranial surface of the proximal half of the diaphysis (1) (modified from Prieto-Márquez 2010, character 277). HL6

133. Pes, prominent extensor processes extending proximodorsally from the dorsal margins of the proximal articulation facets of the distal phalanges: present (0); absent (1). HL7

134. Pes, morphology of unguals on digits II-IV: dorsoventrally flattened, but elongate and pointed (0); dorsoventrally flattened and elongate, but with blunt truncated tips (1); hoof-like shape (2) (modified from Norman 2002, character 67). HL8

**References**

Barrett, P. M., Butler, R. J., Twitchett, R. J., and Hutt, S. 2011. New material of *Valdosaurus*

*canaliculatus* (Ornithischia: Ornithopoda) from the Lower Cretaceous of southern England. *Special Papers in Palaeontology* 86: 131-163.

Barrett, P. M., Butler, R. J., Wang X.-L., and Xu X. 2009. Cranial anatomy of the iguanodontoid ornithopod *Jinzhousaurus yangi* from the Lower Cretaceous Yixian Formation of China. *Acta Palaeontologica Polonica* 54: 35-48.

Boyd, C. A. and Pagnac, D. C. 2015. Insight on the anatomy, systematic relationships, and age of the Early Cretaceous ankylopollexian dinosaur *Dakotadon lakotaensis*. *PeerJ* 3: e1263.

Brill, K. and Carpenter, K. 2006. A description of a new ornithopod from the Lytle Member of the Purgatoire Formation (Lower Cretaceous) and a reassessment of the skull of *Camptosaurus*. In *Horns and Beaks: Ceratopsian and Ornithopod Dinosaurs* (ed. K. Carpenter), pp. 49-67. Bloomington: Indiana University Press.

Brown, B. 1914. *Corythosaurus casuarius*, a new crested dinosaur from the Belly River Cretaceous, with provisional classification of the family Trachodontidae. *Bulletin of the American Museum of Natural History* 33: 559-564.

Brown, B. 1916. *Corythosaurus casuarius*: skeleton, musculature and epidermis. *Bulletin of the American Museum of Natural History* 35: 709-716.

Campione, N. E. and Evans, D. C. 2011. Cranial growth and variation in edmontosaurs (Dinosauria: Hadrosauridae): Implications for latest Cretaceous megaherbivore diversity in North America. *PLoS ONE* 6(9): e25186.

Carpenter, K. and Ishida, Y. 2010. Early and “middle” Cretaceous iguanodonts in time and space. *Journal of Iberian Geology* 36: 145-164.

Carpenter, K., Dilkes, D., and Weishampel, D. B. 1995. The dinosaurs of the Niobrara Chalk Formation (Upper Cretaceous, Kansas). *Journal of Vertebrate Paleontology* 15: 275-297.

Dalla Vecchia, F. M. 2009a *Telmatosaurus* and the other hadrosauroids of the Cretaceous European Archipelago. An update. *Natura Nascosta* 39: 1-18.

Dalla Vecchia, F. M. 2009b. *Tethyshadros insularis*, a new hadrosauroid dinosaur (Ornithischia) from the Upper Cretaceous of Italy. *Journal of Vertebrate Paleontology* 29: 1100-1116.

Gasca, J. M., Canudo, J. I., and Moreno-Azanza, M. 2014. On the diversity of Iberian iguanodont dinosaurs: new fossils from the lower Barremian, Teruel province, Spain. *Cretaceous Research* 50: 264-272.

Gasca, J. M., Moreno-Azanza, M., Ruiz-Omeñaca, J. I., and Canudo, J. I. 2015. New material and phylogenetic position of the basal iguanodont dinosaur *Delapparentia turolensis* from the Barremian (Early Cretaceous) of Spain. *Journal of Iberian Geology* 41: 57-70.

Gasulla, J. M., Escaso, F., Narváez, I., Ortega, F., and Sanz, J. L. 2015. A new sail-backed styracosternan (Dinosauria: Ornithopoda) from the Early Cretaceous of Morella, Spain. *PLoS ONE* 10(12): e0144167.

Gates, T. A. and Sampson, S. D. 2007. A new species of *Gryposaurus* (Dinosauria:

Hadrosauridae) from the late Campanian Kaiparowits Formation, southern Utah, USA. *Zoological Journal of the Linnean Society* 151: 351-376.

Gilmore, C. W. 1909. Osteology of the Jurassic reptile *Camptosaurus*, with a revision of the species of the genus, and descriptions of two new species. *Proceedings of the United States National Museum* 36: 197-332.

Gilmore, C. W. 1933. On the dinosaurian fauna of the Iren Dabasu Formation. *Bulletin of the American Museum of Natural History* 67: 23-78.

Godefroit, P., Codrea, V., and Weishampel, D. B. 2009. Osteology of *Zalmoxes shqiperorum*

(Dinosauria, Ornithopoda), based on new specimens from the Upper Cretaceous of Nǎlaţ-Vad (Romania). *Geodiversitas* 31: 525-553.

Godefroit, P., Escuillié, F., Bolotsky, Y. L., and Lauters, P. 2012. A new basal hadrosauroid dinosaur from the Upper Cretaceous of Kazakhstan. In Bernissart Dinosaurs and Early Cretaceous Terrestrial Ecosystems (ed. P. Godefroit), pp. 334-358. Bloomington: Indiana University Press.

Godefroit, P., Dong Z.-M., Bultynck, P., Li H., and Feng L. 1998. New *Bactrosaurus* (Dinosauria: Hadrosauroidea) material from Iren Dabasu (Inner Mongolia, P. R. China). *Bulletin de l’Institut Royal des Sciences Naturelles de Belgique, Sciences de la Terra* 68 (Supplement): 3-70.

Head, J. J. 1998. A new species of basal hadrosaurid (Dinosauria, Ornithischia) from the Cenomanian of Texas. *Journal of Vertebrate Paleontology* 18: 718-738.

Head, J. J. 2001. A reanalysis of the phylogenetic position of *Eolambia caroljonesa* (Dinosauria, Iguanodontia). *Journal of Vertebrate Paleontology* 21: 392-396.

Hooley, R. W. 1925. On the skeleton of *Iguanodon atherfieldensis* sp. nov., from the Wealden Shales of Atherfield (Isle of Wight). *Quarterly Journal of the Geological Society of London* 81: 1-61.

Horner, J. R., Weishampel, D. B., and Forster, C. A. 2004. Hadrosauridae. In *The Dinosauria:*

*Second Edition* (eds. D. B. Weishampel, P. Dodson, and H. Osmólska), pp. 438-463. Berkeley: University of California Press.

Kirkland, J. I. 1998. A new hadrosaurid from the upper Cedar Mountain Formation (Albian-Cenomanian: Cretaceous) of eastern Utah - the oldest known hadrosaurid (lambeosaurine?). In *Lower and Middle Cretaceous Terrestrial Ecosystems* (eds. S. G. Lucas, J. I. Kirkland, and J. W. Estep). *New Mexico Museum of Natural History and Science Bulletin* 14: 283-295.

Kobayashi, Y. and Azuma, Y. 2003. A new iguanodontian (Dinosauria: Ornithopoda) from the Lower Cretaceous Kitadani Formation of Fukui Prefecture, Japan. *Journal of Vertebrate Paleontology* 23: 166-175.

Lu, J. 1997. A new Iguanodontidae (*Probactrosaurus mazongshanensis* sp. nov.) from Mazongshan Area, Gansu Province, China. In *Sino-Japanese Silk Road Dinosaur Expedition* (ed. Z. Dong), pp. 27-47. Beijing: China Ocean Press.

McDonald, A. T. 2011. The taxonomy of species assigned to *Camptosaurus* (Dinosauria: Ornithopoda). *Zootaxa* 2783: 52-68.

McDonald, A. T. 2012. The status of *Dollodon* and other basal iguanodonts (Dinosauria: Ornithischia) from the Lower Cretaceous of Europe. *Cretaceous Research* 33: 1-6.

McDonald, A. T., Wolfe, D. G., and Kirkland, J. I. 2010a. A new basal hadrosauroid (Dinosauria: Ornithopoda) from the Turonian of New Mexico. *Journal of Vertebrate Paleontology* 30: 799-812.

McDonald, A. T., Kirkland, J. I., DeBlieux, D. D., Madsen, S. K., Cavin, J., Milner, A. R. C., and Panzarin, L. 2010b. New basal iguanodonts from the Cedar Mountain Formation of Utah and the evolution of thumb-spiked dinosaurs. *PLoS ONE* 5(11): e14075.

McDonald, A. T., Bird, J., Kirkland, J. I., and Dodson, P. 2012a. Osteology of the basal hadrosauroid *Eolambia caroljonesa* (Dinosauria: Ornithopoda) from the Cedar Mountain Formation of Utah. *PloS ONE* 7(10): e45712.

McDonald, A. T., Espílez, E., Mampel, L., Kirkland, J. I., and Alcalá, L. 2012b. An unusual new basal iguanodont (Dinosauria: Ornithopoda) from the Lower Cretaceous of Teruel, Spain. *Zootaxa* 3595: 61-76.

McDonald, A. T., Maidment, S. C. R., Barrett, P. M., You, H.-L., and Dodson, P. 2014. Osteology of the basal hadrosauroid *Equijubus normani* (Dinosauria, Ornithopoda) from the Early Cretaceous of China. In *Hadrosaurs* (eds. D. A. Eberth and D. C. Evans), pp. 44-72. Bloomington: Indiana University Press.

Mo J., Zhao Z., Wang W., and Xu X. 2007. The first hadrosaurid dinosaur from southern China. *Acta Geologica Sinica – English Edition* 81: 550-554.

Norman, D. B. 1980. On the ornithischian dinosaur *Iguanodon bernissartensis* from Belgium. *Mémoires Institut Royal des Sciences Naturelles de Belgique* 178: 1-103.

Norman, D. B. 1986. On the anatomy of *Iguanodon atherfieldensis* (Ornithischia: Ornithopoda). *Bulletin de l’Institut Royal des Sciences Naturelles de Belgique* 56: 281-372.

Norman, D. B. 1993. Gideon Mantell’s “Mantel-piece”: the earliest well-preserved ornithischian dinosaur. *Modern Geology* 18: 225-245.

Norman, D. B. 1998. On Asian ornithopods (Dinosauria: Ornithischia). 3. A new species of iguanodontid dinosaur. *Zoological Journal of the Linnean Society* 122: 291-348.

Norman, D. B. 2002. On Asian ornithopods (Dinosauria: Ornithischia). 4. *Probactrosaurus* Rozhdestvensky, 1966. *Zoological Journal of the Linnean Society* 136: 113-144.

Norman, D. B. 2004. Basal Iguanodontia. In *The Dinosauria: Second Edition* (eds. D. B. Weishampel, P. Dodson, and H. Osmólska), pp. 413-437. Berkeley: University of California Press.

Norman, D. B. 2010. A taxonomy of iguanodontians (Dinosauria: Ornithopoda) from the lower Wealden Group (Cretaceous: Valanginian) of southern England. *Zootaxa* 2489: 47-66.

Norman, D. B. 2011. On the osteology of the lower Wealden (Valanginian) ornithopod *Barilium dawsoni* (Iguanodontia: Styracosterna). *Special Papers in Palaeontology* 86: 165-194.

Norman, D. B. 2012. Iguanodontian taxa (Dinosauria: Ornithischia) from the Lower Cretaceous of England and Belgium. In Bernissart Dinosaurs and Early Cretaceous Terrestrial Ecosystems (ed. P. Godefroit), pp. 174-212. Bloomington: Indiana University Press.

Norman, D. B. 2014. On the history, osteology, and systematic position of the Wealden (Hastings Group) dinosaur *Hypselospinus fittoni* (Iguanodontia: Styracosterna). *Zoological Journal of the Linnean Society* 173: 92-189.

Ostrom, J. H. 1961. Cranial morphology of the hadrosaurian dinosaurs of North America. *Bulletin of the American Museum of Natural History* 122: 33-186.

Prieto-Márquez, A. 2010. Global phylogeny of hadrosauridae (Dinosauria: Ornithopoda) using parsimony and Bayesian methods. *Zoological Journal of the Linnean Society* 159: 435-502.

Prieto-Márquez, A. 2011. Revised diagnoses of *Hadrosaurus foulkii* Leidy 1858 (the type genus and species of Hadrosauridae Cope, 1869) and *Claosaurus agilis* Marsh, 1872 (Dinosauria: Ornithopoda) from the Late Cretaceous of North America. *Zootaxa* 2765: 61-68.

Prieto-Márquez, A. 2014. A juvenile *Edmontosaurus* from the late Maastrichtian (Cretaceous) of North America: Implications for ontogeny and phylogenetic inference in saurolophine dinosaurs. *Cretaceous Research* 50: 282-303.

Prieto-Márquez, A. and Norell, M. A. 2010. Anatomy and relationships of *Gilmoreosaurus mongoliensis* (Dinosauria: Hadrosauroidea) from the Late Cretaceous of Central Asia. *American Museum Novitates* 3694: 1-49.

Prieto-Márquez, A., Weishampel, D. B., and Horner, J. R. 2006a. The dinosaur *Hadrosaurus foulkii*, from the Campanian of the East Coast of North America, with a reevaluation of the genus. *Acta Palaeontologica Polonica* 51: 77-98.

Prieto-Márquez, A., Gaete, R., Rivas, G., Galobart, À., and Boada, M. 2006b. Hadrosauroid dinosaurs from the Late Cretaceous of Spain: *Pararhabdodon isonensis* revisited and *Koutalisaurus kohlerorum*, gen. et sp. nov. *Journal of Vertebrate Paleontology* 26: 929-943.

Ramírez-Velasco, A. A., Benammi, M., Prieto-Márquez, A., Ortega, J. A., and Hernández-Rivera, R. 2012. *Huehuecanauhtlus tiquichensis*, a new hadrosauroid dinosaur (Ornithischia: Ornithopoda) from the Santonian (Late Cretaceous) of Michoacán, Mexico. *Canadian Journal of Earth Sciences* 49: 379-395.

Ruiz-Omeñaca, J. I. 2011. *Delapparentia turolensis* nov. gen et sp., a new iguanodontoid dinosaur (Ornithischia: Ornithopoda) from the Lower Cretaceous of Galve (Spain). *Estudios Geológicos* 67: 83-110.

Shibata, M. and Azuma, Y. 2015. New basal hadrosauroid (Dinosauria: Ornithopoda) from the Lower Cretaceous Kitadani Formation, Fukui, central Japan. *Zootaxa* 3914: 421-440.

Shibata, M., Jintasakul, P., Azuma, Y., and You, H.-L. 2015. A new basal hadrosauroid dinosaur from the Lower Cretaceous Khok Kruat Formation in Nakhon Ratchasima Province, northeastern Thailand. *PLoS ONE* 10(12): e0145904.

Sues, H.-D. and Averianov, A. 2009. A new basal hadrosauroid dinosaur from the Late Cretaceous of Uzbekistan and the early radiation of duck–billed dinosaurs. *Proceedings of the Royal Society B* 276: 2549-2555.

Taquet, P. 1976. Ostéologie d’*Ouranosaurus nigeriensis*, Iguanodontide du Crétacé Inférieur du Niger. *Géologie et Paléontologie du Gisement de Gadoufaoua (Aptien du Niger)*, Chapitre III. pp. 57-168.

Taquet, P. and Russell, D. A. 1999. A massively-constructed iguanodont from Gadoufaoua, Lower Cretaceous of Niger. *Annales de Paléontologie* 85: 85-96.

Tsogtbaatar, K., Weishampel, D. B., Evans, D. C., and Watabe, M. 2014. A new hadrosauroid (*Plesiohadros djadokhtaensis*) from the Late Cretaceous Djadokhtan fauna of southern Mongolia. In *Hadrosaurs* (eds. D. A. Eberth and D. C. Evans), pp. 108-135. Bloomington: Indiana University Press.

Verdú, F. J., Royo-Torres, R., Cobos, A., and Alcalá, L. 2015. Perinates of a new species of *Iguanodon* (Ornithischia: Ornithopoda) from the lower Barremian of Galve (Teruel, Spain). *Cretaceous Research* 56: 250-264.

Wang, R.-F., You, H.-L., Xu, S.-C., Wang, S.-Z., Yi, J., Xie, L.-J., Jia, L., and Li, Y.-X. 2013. A new hadrosauroid dinosaur from the early Late Cretaceous of Shanxi Province, China. *PLoS ONE* 8(10): e77058.

Wang, X., Pan, R., Butler, R. J., and Barrett, P. M. 2011. The postcranial skeleton of the iguanodontian ornithopod *Jinzhousaurus yangi* from the Lower Cretaceous Yixian Formation of western Liaoning, China. *Earth and Environmental Science Transactions of the Royal Society of Edinburgh* 101: 135-159.

Weishampel, D. B. and Bjork, P. R. 1989. The first indisputable remains of *Iguanodon* (Ornithischia: Ornithopoda) from North America: *Iguanodon lakotaensis*, sp. nov. *Journal of Vertebrate Paleontology* 9: 56-66.

Weishampel, D. B., Norman, D. B., and Grigorescu, D. 1993. *Telmatosaurus transsylvanicus* from the Late Cretaceous of Romania: the most basal hadrosaurid dinosaur. *Palaeontology* 36: 361-385.

Weishampel, D. B., Jianu, C.-M., Csiki, Z., and Norman, D. B. 2003. Osteology and phylogeny of *Zalmoxes* (n. g.), an unusual euornithopod dinosaur from the latest Cretaceous of Romania. *Journal of Systematic Palaeontology* 1: 65-123.

Wiman, C. 1929. Die Kreide-dinosaurier aus Shantung. *Palaeontologia Sinica* 6: 1-63.

Winkler, D. A., Murry, P. A., and Jacobs, L. L. 1997. A new species of *Tenontosaurus*

(Dinosauria: Ornithopoda) from the Early Cretaceous of Texas. *Journal of Vertebrate Paleontology* 17: 330­­-348.

Wu W. and Godefroit, P. 2012. Anatomy and relationships of *Bolong yixianensis*, an Early Cretaceous iguanodontoid dinosaur from western Liaoning, China. In Bernissart Dinosaurs and Early Cretaceous Terrestrial Ecosystems (ed. P. Godefroit), pp. 292-333. Bloomington: Indiana University Press.

Xing, H., Wang, D., Han, F., Sullivan, C., Ma, Q., He, Y., Hone, D. W. E., Yan, R., Du, F., and Xu, X. 2014. A new basal hadrosauroid dinosaur (Dinosauria: Ornithopoda) with transitional features from the Late Cretaceous of Henan Province, China. *PLoS ONE* 9(6): e98821.

Xu X., Zhao X. J., Lu J.-C., Huang W.-B., Li Z.-Y., and Dong Z.-M. 2000. A new iguanodontian from Sangping Formation of Neixiang, Henan and its stratigraphical implications. *Vertebrata PalAsiatica* 38: 176-191.

You H.-L. and Li D.-Q. 2009. A new basal hadrosauriform dinosaur (Ornithischia: Iguanodontia) from the Early Cretaceous of northwestern China. *Canadian Journal of Earth Sciences* 46: 949-957.

You H., Ji Q., and Li D. 2005. *Lanzhousaurus magnidens* gen. et sp. nov. from Gansu Province, China: the largest-toothed herbivorous dinosaur in the world. *Geological Bulletin of China* 24: 785-794.

You H., Li D., and Liu W. 2011. A new hadrosauriform dinosaur from the Early Cretaceous of Gansu Province, China. *Acta Geologica Sinica-English Edition* 85: 51-57.

You, H.-L., Li, D.-Q., and Dodson, P. 2014. *Gongpoquansaurus mazongshanensis* (Lü, 1997) comb. nov. (Ornithischia: Hadrosauroidea) from the Early Cretaceous of Gansu Province, northwestern China. In *Hadrosaurs* (eds. D. A. Eberth and D. C. Evans), pp. 73-76. Bloomington: Indiana University Press.

You H., Ji Q., Li J., and Li Y. 2003a. A new hadrosauroid dinosaur from the mid-Cretaceous of Liaoning, China. *Acta Geologica Sinica-English Edition* 77: 148-154.

You, H., Luo, Z., Shubin, N. H., Witmer, L. M., Tang, Z., and Tang, F. 2003b. The earliest-known duck-billed dinosaur from deposits of late Early Cretaceous age in northwest China and hadrosaur evolution. *Cretaceous Research* 24: 347-355.

Zheng, W., Jin, X., Shibata, M., and Azuma, Y. 2014. An early juvenile specimen of *Bolong yixianensis* (Ornithopoda: Iguanodontia) from the Lower Cretaceous of Ningcheng County, Nei Mongol, China. *Historical Biology* 26: 236-251.

**List of Synapomorphies**

Synapomorphies that unite nodes in the 50% Majority Rule consensus tree. Node numbers correspond to those in Figure 15 of the main text.

| **Nodes** | **Synapomorphies** |
| --- | --- |
| 1 | 102^1^, 112^1^ |
| 2 | 52^1^, 56^1^, 57^1^ |
| 3 | 14^1^, 77^1^ |
| 4 | 5^1^, 16^1^, 17^1^, 23^1^ |
| 5 | 21^1^, 37^1^, 39^1^, 50^1^, 103^1^, 128^1^ |
| 6 | 13^1^, 61^1^, 72^1^, 75^1^, 76^1^ |
| 7 | 3^1^, 15^1^, 28^1^, 52^2^ |
| 8 | 21^0^, 44^0^, 66^1^, 69^1^, 88^1^, 93^2^ |
| 9 | 2^1^, 13^0^, 18^1^, 119^2^ |
| 10 | 1^0^, 22^1^, 108^0^ |
| 11 | 40^1^ |
| 12 | 38^3^, 39^0^, 125^1^ |
| 13 | 12^0^ |
| 14 | 22^1^, 42^0^, 50^2^, 116^2^, 126^1^ |
| 15 | 28^0^, 68^0^ |
| 16 | 82^2^, 85^2^ |
| 17 | 73^1^, 77^2^, 80^1^ |
| 18 | 104^1^, 107^1^, 117^2^, 134^2^ |
| 19 | 11^1^, 54^1^ |
| 20 | 35^2^, 59^1^ |
| 21 | 37^2^, 55^1^ |
| 22 | 97^1^ |
| 23 | 119^2^ |
| 24 | 69^1^ |
| 25 | 3^2^, 6^1^, 8^1^, 66^1^, 115^2^, 130^2^ |
| 26 | 57^2^ |
| 27 | 48^0^, 71^0^, 74^1^, 106^1^ |
| 28 | 7^1^, 34^1^ |
| 29 | 16^0^ |
| 30 | 4^1^, 16^2^, 102^0^ |
| 31 | 72^0^, 73^0^ |
| 32 | 81^1^, 82^4^ |
| 33 | 102^2^, 109^2^ |
| 34 | 97^1^, 110^1^ |
