# Supplementary material for: Anatomy, taphonomy, and phylogenetic implications of a new specimen of Eolambia caroljonesa (Dinosauria: Ornithopoda) from the Cedar Mountain Formation, Utah, USA
Source: PLoS One. 2017 May 10;12(5):e0176896. doi: 10.1371/journal.pone.0176896 (PMC5425030; doi:10.1371/journal.pone.0176896)
Supplement: S1 Table — Measurements of select anatomical features of FMNH PR 3847. (DOC) [file pone.0176896.s005.doc]

**S1 Table. Table of Measurements.** Measurements of FMNH PR 3847. Numbered elements correspond to the numbers in Figure 1 of the paper.

| **Elements** | **Measurements (in centimeters)** |
| --- | --- |
| 1. Middle cervical neural arch |  |
| Right prezygapophysis, length | 3.0 |
| Left prezygapophysis, length | 3.0 |
| Right postzygapophysis, length | 5.1 |
| Left postzygapophysis, length | 5.2 |
|  |  |
| 2. Cervical centrum |  |
| Craniocaudal length | 4.2 |
| Cranial face, height | 3.7 |
| Cranial face, width | 6.3 |
| Caudal face, height | 4.4 |
| Caudal face, width | 6.8 |
|  |  |
| 4. Caudal cervical vertebra, right half |  |
| Centrum, craniocaudal length | 6.2 |
| Centrum, cranial face, height | 6.6 |
| Centrum, caudal face, height | 7.1 |
|  |  |
| 5. Cranial dorsal vertebra (D1) |  |
| Centrum, craniocaudal length | 6.0 |
| Centrum, cranial face, height | 5.5 |
| Centrum, cranial face, width | 7.2 |
| Centrum, caudal face, height | 6.3 |
| Centrum, caudal face, width | 8.6 |
| Right postzygapophysis, length | 8.1 |
|  |  |
| 6. Cranial dorsal neural arch (D3 or D4) |  |
| Right prezygapophysis, length | 3.2 |
| Left prezygapophysis, length | 3.2 |
| Width across transverse processes | 21.8 |
|  |  |
| 7. Caudal dorsal neural arch (D12 or D13) |  |
| Right prezygapophysis, length | 4.1 |
| Left prezygapophysis, length | 4.2 |
| Right postzygapophysis, length | 3.5 |
| Width across transverse processes | 24.0 |
|  |  |
| 8. Caudal dorsal centrum (D12) |  |
| Craniocaudal length | 7.3 |
| Cranial face, height | 8.0 |
| Cranial face, width | 6.8 |
| Caudal face, height | 7.7 |
| Caudal face, width | 7.5 |
|  |  |
| 9. Caudal dorsal centrum (D13) |  |
| Craniocaudal length | 6.5 |
| Cranial face, height | 8.6 |
| Cranial face, width | 8.4 |
| Caudal face, height | 9.1 |
| Caudal face, width | 10.2 |
|  |  |
| 10. Caudal dorsal centrum (D14) |  |
| Craniocaudal length | 7.5 |
| Cranial face, height | 6.8 |
| Cranial face, width | 7.4 |
| Caudal face, height | 6.8 |
| Caudal face, width | 6.5 |
|  |  |
| 11. Caudal dorsal centrum (D15) |  |
| Craniocaudal length | 4.2 |
| Cranial face, height | 9.8 |
| Cranial face, width | 8.2 |
| Caudal face, height | 10.1 |
| Caudal face, width | 9.5 |
|  |  |
| 12. Caudal dorsal centrum (D16) |  |
| Craniocaudal length | 6.6 |
| Cranial face, height | 7.7 |
| Cranial face, width | 9.7 |
| Caudal face, height | 8.8 |
| Caudal face, width | 10.9 |
|  |  |
| 13. Dorsal rib |  |
| Proximodistal length | 84.3 |
| Capitulum, mediolateral length | 10.4 |
| Capitulum, dorsoventral depth | 4.0 |
|  |  |
| 14. Proximal caudal centrum |  |
| Craniocaudal length | 6.4 |
| Cranial face, height | 9.5 |
| Cranial face, width | 9.1 |
| Caudal face, height | 9.0 |
| Caudal face, width | 9.2 |
|  |  |
| 15. Proximal caudal centrum |  |
| Craniocaudal length | 6.4 |
| Cranial face, height | 9.3 |
| Cranial face, width | 8.7 |
| Caudal face, height | 9.6 |
| Caudal face, width | 8.8 |
|  |  |
| 16. Proximal caudal centrum |  |
| Craniocaudal length | 5.7 |
| Cranial face, height | 8.4 |
| Cranial face, width | 8.3 |
| Caudal face, height | 8.1 |
| Caudal face, width | 8.0 |
|  |  |
| 17. Proximal caudal centrum |  |
| Craniocaudal length | 6.7 |
| Cranial face, height | 8.0 |
| Cranial face, width | 8.4 |
| Caudal face, height | 7.5 |
| Caudal face, width | 7.7 |
|  |  |
| 18. Proximal caudal centrum |  |
| Craniocaudal length | 6.4 |
| Cranial face, height | 9.7 |
| Cranial face, width | 7.8 |
|  |  |
| 22. Middle caudal vertebra, centrum |  |
| Craniocaudal length | 6.6 |
| Cranial face, height | 6.1 |
| Cranial face, width | 6.4 |
| Caudal face, height | 6.8 |
| Caudal face, width | 5.9 |
|  |  |
| 23. Middle caudal vertebra, centrum |  |
| Craniocaudal length | 6.8 |
| Cranial face, height | 6.0 |
| Cranial face, width | 7.0 |
| Caudal face, height | 6.6 |
| Caudal face, width | 7.1 |
|  |  |
| 25. Proximal chevron |  |
| Transverse width of proximal end | 2.8 |
|  |  |
| 26. Left ilium |  |
| Total craniocaudal length | 70.8 |
| Preacetabular process, craniocaudal length | ~26 |
| Acetabulum, craniocaudal length | ~20 |
| Postacetabular process, craniocaudal length | ~24 |
|  |  |
| 27. Left pubis, cranial pubic process |  |
| Craniocaudal length | 48.4 |
| Minimum dorsoventral depth of constriction | 9.6 |
| Maximum dorsoventral depth of expansion | ~17 |
